# Genome-wide identification of miRNAs and lncRNAs in *Cajanus cajan*

**DOI:** 10.1186/s12864-017-4232-2

**Published:** 2017-11-15

**Authors:** Chandran Nithin, Amal Thomas, Jolly Basak, Ranjit Prasad Bahadur

**Affiliations:** 1 Computational Structural Biology Lab, Department of Biotechnology, Indian Institute of Technology Kharagpur, Kharagpur, India; 2 Department of Biotechnology, Visva-Bharati, Santiniketan, India; 3 Present address: Molecular and Computational Biology, Department of Biological Sciences, University of Southern California, Los Angeles, California USA

**Keywords:** miRNA, lncRNA, *Cajanus Cajan*, SSR signature, genome-wide analysis

## Abstract

**Background:**

Non-coding RNAs (ncRNAs) are important players in the post transcriptional regulation of gene expression (PTGR). On one hand, microRNAs (miRNAs) are an abundant class of small ncRNAs (~22nt long) that negatively regulate gene expression at the levels of messenger RNAs stability and translation inhibition, on the other hand, long ncRNAs (lncRNAs) are a large and diverse class of transcribed non-protein coding RNA molecules (> 200nt) that play both up-regulatory as well as down-regulatory roles at the transcriptional level. *Cajanus cajan,* a leguminosae pulse crop grown in tropical and subtropical areas of the world, is a source of high value protein to vegetarians or very poor populations globally. Hence, genome-wide identification of miRNAs and lncRNAs in *C. cajan* is extremely important to understand their role in PTGR with a possible implication to generate improve variety of crops.

**Results:**

We have identified 616 mature miRNAs in *C. cajan* belonging to 118 families, of which 578 are novel and not reported in MirBase21. A total of 1373 target sequences were identified for 180 miRNAs. Of these, 298 targets were characterized at the protein level. Besides, we have also predicted 3919 lncRNAs. Additionally, we have identified 87 of the predicted lncRNAs to be targeted by 66 miRNAs.

**Conclusions:**

miRNA and lncRNAs in plants are known to control a variety of traits including yield, quality and stress tolerance. Owing to its agricultural importance and medicinal value*,* the identified miRNA, lncRNA and their targets in *C. cajan* may be useful for genome editing to improve better quality crop. A thorough understanding of ncRNA-based cellular regulatory networks will aid in the improvement of *C. cajan* agricultural traits.

**Electronic supplementary material:**

The online version of this article (10.1186/s12864-017-4232-2) contains supplementary material, which is available to authorized users.

## Background

*Cajanus cajan* is a major source of protein for the poor communities of many tropical and subtropical regions of the world [1]. The high protein and carbohydrate contents make it not only important to the human diet, but also suitable as high protein feed and fodder ingredient to livestock [2]. With its greater tolerance to heat, drought, and low soil fertility, *C. cajan* is a valuable component of low external input agricultural farming systems where the farmers have scarcity of resources [3–6]. *C. cajan* is a good source of sulphur containing amino acids, crude fibre, iron, sulphur, calcium, potassium, manganese and water soluble vitamins especially thiamine, riboflavin and niacin [7, 8]. In addition to these, several flavonoids, isoflavonoids, tannins and protein fractions have been isolated from the different parts of *C. cajan* and their medicinal uses have been established [9].

The ncRNAs are a wide class of non-coding RNAs that are transcribed but not translated and play a major role in post-transcriptional gene regulations. Based on their length, ncRNAs are generally classified into small non-coding RNAs (sncRNAs) and long non-coding RNAs (lncRNAs). MicroRNAs (miRNA) are an abundant class of sncRNAs (~22nt long), which negatively regulate gene expression at the levels of messenger RNAs (mRNAs) stability and translation inhibition. In addition to this, the miRNAs are also known to interact with lncRNAs as well as competing endogenous RNAs (ceRNAs) that de-repress the gene expression. Identification of the various miRNAs and their targets is important in understanding the dynamics of gene regulation and in designing new breeds of crops with higher productivity and better disease resistance. In spite of having immense importance, there are only few studies have ventured into identifying the miRNAs in *C. cajan* [10]. Additionally, miRNAs of *C. cajan* are still missing in miRBase 21 [11].

The miRNAs are known to have sequence conservation and are grouped into various miRNAs families in miRBase. The presence of orthologs and paralogs among miRNA sequences allows the identification of miRNAs by using computational methods starting from the sequence similarity. The mere presence of a sequence match on a genome does not imply that the identified region is a miRNA. miRNA precursor sequences (pre-miRs) are known to have features distinct from other small RNA. The mapping of known miRNAs to the genome followed by extraction and analysis of pre-miRs is an effective strategy in miRNA discovery [10, 12–17]. Various sequence based information as well as structural attributes of the pre-miRs can be useful to establish whether a given match is a miRNA sequence or not. To begin with, the miRNA precursors have a distinct range in which the nucleotide composition falls [18]. The pre-miRNAs also have a distinct pattern in the free energy of folding [19]. The minimal folding energy index (MFEI), which is the free energy associated with folding, normalized per GC content per hundred nucleotides, is used as a parameter in predicting miRNAs. The miRNAs are also shown to have distinct region in the probability distributions of RNA folding measures, namely, normalized Shannon entropy (NQ), normalized base pairing propensity (Npb) and normalized base pairing distance (ND) [16]. Simple sequence repeats (SSRs) are one to six nucleotides long repeat sequences present in the pre-miRs [20], and can be used as a parameter to efficiently predict miRNAs [16].

lncRNAs are a large and diverse class of transcribed non-protein coding RNA molecules with a length of more than 200 nucleotides. The evidence for regulatory role of lncRNAs in important biological processes was first identified during the 1980s from genetic analyses of the *Drosophila* bithorax complex [21]. Compared to the protein coding mRNAs, lncRNAs have certain specific properties, namely, shorter length, lower abundance, restriction to particular tissues or cells and less frequent conservation between species [22]. The lncRNA biogenesis is very similar to protein coding mRNAs but some lncRNAs are transcribed by RNA polymerase III [23]. The lncRNAs also have the post-transcriptional modifications like 5’ capping, splicing and polyadenylation [24]. While most of the lncRNAs are localized within the nucleus, there are a few exceptions that perform functions in the cytosol [25, 26]. The origin of lncRNAs can range from intronic, exonic, intergenic, intragenic, promoter regions, 3’- and 5’- UTRs and enhancer sequences. The transcription of lncRNAs can happen either in sense or in antisense directions [27]. They play both down regulatory as well as up regulatory roles at the transcriptional level. The lncRNAs originating from protein coding loci competes for the RNA polymerase II and other initiation factors or cause the premature termination of elongation complex [28]. The lncRNAs can enhance the accessibility of target site to RNA polymerase and thereby upregulate the gene expression [29]. Some lncRNAs bind to the promoter DNA of target gene, forming a RNA-dsDNA triplex that prevents the preinitiation complex from accessing the target gene promoter [30]. There are also lncRNAs which are reported to regulate the gene expression by inhibiting the RNA polymerase activities or by controlling the subcellular localization of transcription factors [31–33]. In addition to the transcriptional regulation, lncRNAs also play a role in post-transcriptional modulations of mRNA processing. They play role in pre-mRNA alternate splicing, transport, translation and degradation [34]. The lncRNAs can also cause the degradation of target mRNA through the formation of a double stranded RNA duplex, which is processed into endo-siRNAs [35].

In this study, we have identified 616 miRNAs in *C. cajan*, of which 578 are novel and not reported in MirBase21. Besides, we have also predicted 3919 lncRNAs. Additionally, the protein coding genes targeted by many of the miRNAs are identified in this study, facilitating a functional annotation to the predicted miRNAs. Moreover, we have identified the lncRNAs that are targeted by miRNAs. These findings will significantly contribute to the present knowledge of ncRNAs in *C. cajan*, and will enhance our understanding for genome editing and improving the crop varieties in plants.

## Methods

### Dataset collection and preparation

The dataset of known miRNAs and pre-miRs was downloaded from miRBase 21 [36], which consists of 4800 mature and 8480 pre-miRs belonging to 73 species of Viridiplantae. Besides, we have also downloaded the draft genome sequence of *C. cajan* [37]. The coding DNA sequences composed of 21,434 transcriptome assembly contigs, ccTAv2.0, was downloaded from Legume Information system [38]. The protein sequences of Viridiplantae was curated from NCBI [39]. The UniProt proteome, UP000075243, with 47,180 entries was downloaded along with the UniProt-GOA annotation data [40, 41]. The SWISS-PROT database [42] was downloaded for running the BLAST [43] search.

### Prediction of miRNAs

The dataset of known miRNAs was BLAST searched against the genome of *C. cajan*. The BLAST hits with zero to three mismatches with the known miRNAs were selected, and were further used for analysis in the prediction pipeline. The upstream and downstream nucleotides from the BLAST hit was extracted following Nithin, et al. [16], and the protein coding sequences were removed by performing BLASTX with the protein sequences of Viridiplantae. The sequences were selected based on the cut-off value for each of the following parameters: MFEI, NQ, ND, Npb and SSRs [16]. The MFEI value for a sequence of length L was calculated using the adjusted MFE (AMFE), which represents the MFE for 100 nucleotides.

$$ MFEI=\frac{AMFE}{\left(G+C\right)\%}\kern0.5em \mathrm{and}\kern0.5em AMFE=-\frac{MFE}{L}\times 100 $$

The genRNAstats program [19] was used to calculate the NQ, ND and Npb for all known pre-miRs of Viridiplantae. Npb is the measure of total number of base pairs present in the RNA secondary structure per length of the sequence, and the value can range from 0.0 (no base-pairs) to 0.5 (L/2 base-pairs) [44]. The base-pairing probability distribution (BPPD) per base in a sequence were measured using NQ [45], while the base-pair distance for all the pair of structures were measured using ND [46]. Both the parameters ND and NQ were calculated from the MaCaskill base pair probability p_ij_ between the two bases, i and j:

$$ {\displaystyle \begin{array}{c}\mathrm{where}\kern0.5em {p}_{ij}=\sum \limits_{S_{\alpha}\in S(s)}P\left({S}_{\alpha}\right){\delta}_{ij}\\ {}P\left({S}_{\alpha}\right)=\frac{e\frac{-{E}_{\alpha }}{RT}}{\sum \limits_{S_{\alpha}\in S(s)}e\frac{-{E}_{\alpha }}{RT}}\\ {}\mathrm{and}\kern0.5em {\delta}_{ij}=\left\{\begin{array}{l}1,\kern0.5em {\mathrm{x}}_{\mathrm{i}}\kern0.5em \mathrm{pairs}\kern0.5em {\mathrm{x}}_{\mathrm{j}}\\ {}0,\kern1em \mathrm{otherwise}\end{array}\right.\\ {} NQ=-\frac{1}{L}\sum \limits_{i<j}{p}_{ij}{\log}_2\left({p}_{ij}\right)\kern1em \mathrm{and}\  ND=\frac{1}{L}\sum \limits_{i<j}{p}_{ij}\left(1-{p}_{ij}\right)\end{array}} $$

**Fig. 1:**
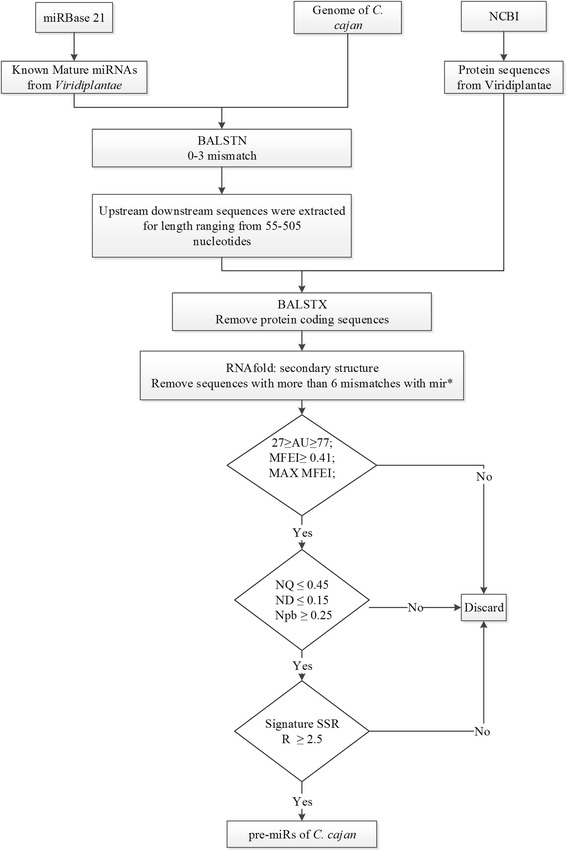
Schematic representation of the methodology followed in the prediction of pre-miRs of *C. cajan*

The signature SSRs for different miRNA families at window size of three were taken from Nithin, et al. [16]. The conserved SSR signatures were normalized per 100 nucleotides (R). The pipeline followed in the prediction of pre-miRs is depicted in Fig. 1.

### Prediction of lncRNAs

**Fig. 2:**
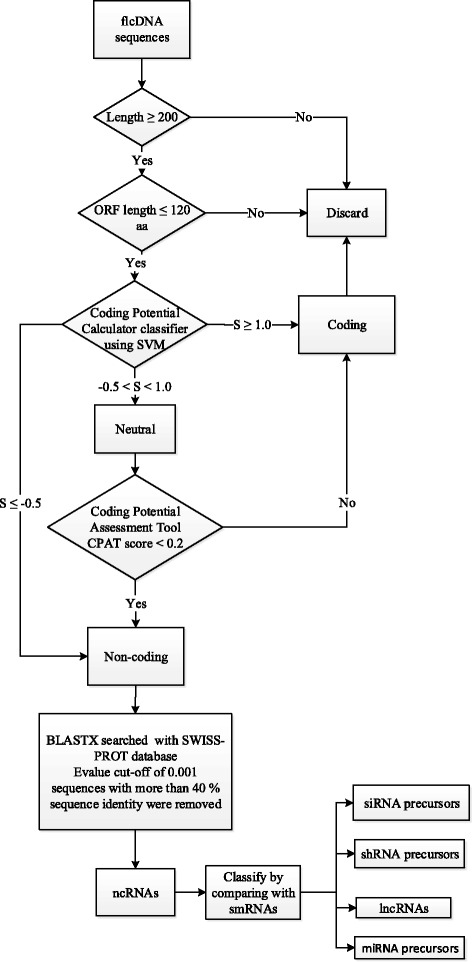
Schematic representation of the methodology followed in the prediction of lncRNAs of *C. cajan*

The coding DNA sequences (CDS) of *C. cajan* were used as the starting point for the prediction of lncRNAs. The CDS with length greater than 200 nucleotides [47] were retained and the ORFs were computed using the EMBOSS getorf standalone [48]. The ORFs with length less than 120 amino acids were retained for further analysis [47]. The coding potential for the sequences were checked by two different algorithms: Coding Potential Calculator (CPC), developed on support vector machine [49], and Coding Potential Assessment Tool (CPAT), which is an alignment-free algorithm [50]. Based on CPC score (S), sequences were classified into non-coding (S ≤ -0.5), neutral (-0.5 < S < 1.0) and coding (S ≥ 1.0) [49]. The sequences classified as neutral were further checked by CPAT. Sequences having CPAT score < 0.2 were classified as ncRNAs [51]. The sequences were further searched using BLASTX [52] against the SWISS-PROT database [42] with an e-value cut-off of 0.001. Sequences with more than 40 % identity were removed, and the remaining sequences were selected as lncRNAs. The pipeline followed for the prediction is represented as a flowchart in Fig. 2.

### Prediction of miRNA targets

The targets for mature miRNAs were predicted using psRNATarget server [53] by submitting the mature miRNAs as query and the CDS sequences of *C. cajan* as subject. To reduce the number of false predictions, the maximum expectation threshold was set to a stringent value of 2.0. The cut-off length of nucleotides for complementarity scoring, hspsize [54], was set as the length of the mature miRNAs. The maximum energy of unpairing (UPE) the target site was set as 25 kcal [54]. The flanking length around the target site was selected as 17 nucleotides upstream and 13 nucleotides downstream [55]. Due to the variable length of the mature miRNAs, the sequence range of the central mismatch was adjusted as described by Nithin, et al. [16]. To predict the function of the target sequences, the sequences were mapped to the UniProt proteome. The miRNAs, targeting the lncRNAs, were predicted by submitting the mature miRNAs as query and the lncRNAs of *C. cajan* as subject following the same pipeline. The interaction networks of miRNAs with target mRNAs were constructed using Cystoscape [56].

## Results and Discussion

### Identification of miRNAs

In this study, we have identified 616 miRNAs from the genome of *C. cajan* by following the prediction pipeline explained in the ‘Materials and Methods’ section. The method has been computationally validated by predicting the miRNAs of model plants *Arabidopsis thaliana* and *Glycine max*. In both the cases, we obtained high specificity and sensitivity [16]. Moreover, we experimentally validated 97 miRNAs, predicted using the pipeline, in a small RNA library prepared from *P. vulgaris* cv. Anupam [16]. Both *G. max* and *P. vulgaris* are members of Fabaceae family and are phylogenetically closely related species of *C. cajan*. Hence, we believe that our hypothesis for prediction of miRNAs and their targets will also holds for *C. cajan.*

The known miRNAs of Viridiplantae were BLAST searched against the genome of *C. cajan* with an e-value cut-off of 1000, allowing zero to three mismatches*.* The mismatches permitted during the BLAST search allows the identification of miRNAs, which are identical to known miRNAs but novel to the plant species that are not reported in the miRBase. From the BLAST search, a total of 1831779306 sequences, that do not code for proteins were extracted with all possible lengths. In case of multiple sequences resulting from a single BLAST hit fulfilling the criteria, the one with the maximum MFEI and the maximum R was retained. A total of 616 miRNAs belonging to 341 miRNA families were identified by the prediction pipeline (Additional file 1: Table S1). A previous study by Kompelli, et al. [10] had identified only 142 miRNAs in *C. cajan*. This lower number of predicted miRNA may be due to the fact that they have used a smaller search space in identifying miRNAs. Of the miRNAs identified in this study, 578 are novel with respect to both plant miRNAs available in miRBase 21 as well as those identified by Kompelli, et al. [10].

**Fig. 3:**
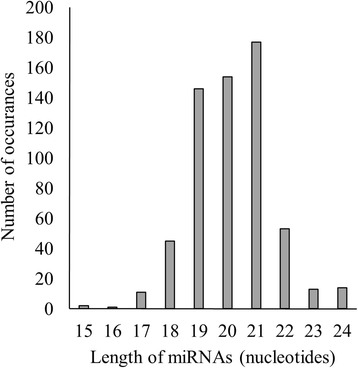
Distribution of the length of miRNAs in *C. cajan*

**Fig. 4:**
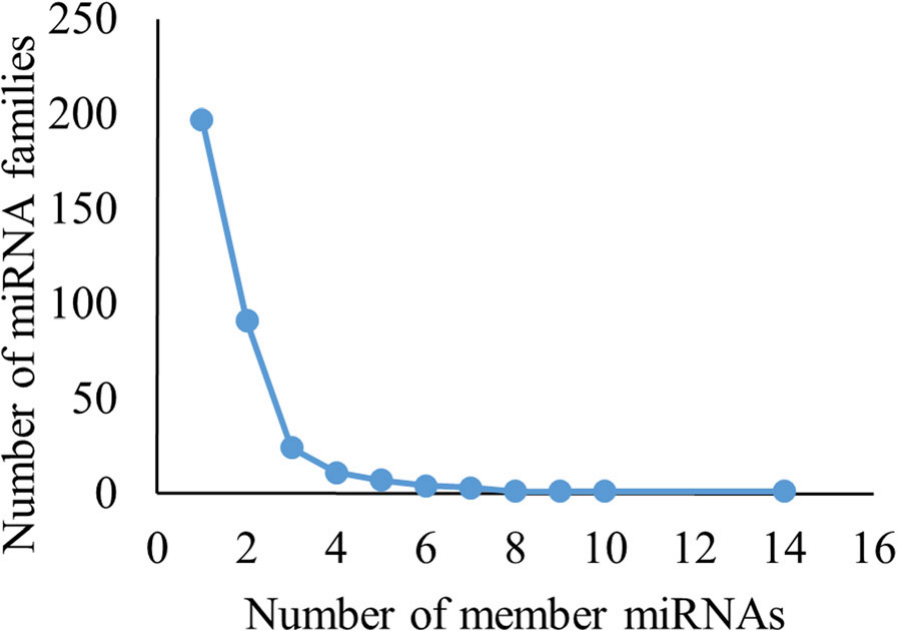
Frequency distribution of miRNAs across the miRNA families in *C. cajan*

**Fig. 5:**
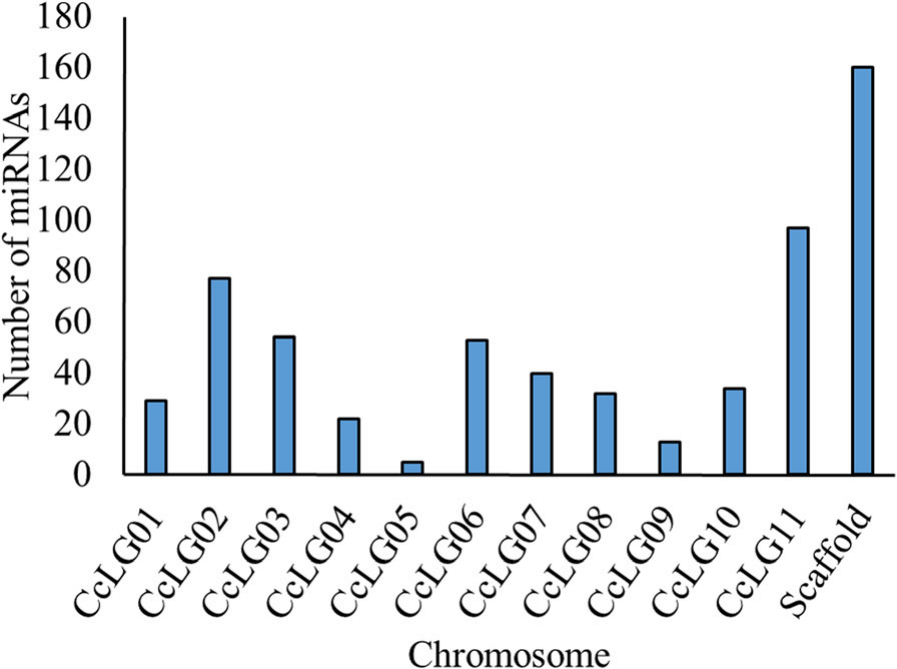
Distribution of miRNAs across different chromosomes of *C. cajan*

The length of mature miRNAs of *C. cajan* varies from 15 to 24 nucleotides (nt) with an average of 20 nt (s.d. is ±1.4). Majority of them (93 %) falls within the range of 18 to 22 nt. Figure 3 shows the distribution of length of miRNAs identified in this study. MiRBase 21 has classified the 4800 plant miRNAs into 2290 families. The 616 miRNAs identified in this study belongs to 341 different families (Table 1). The distribution of miRNAs across various families is highly heterogeneous. Majority (85 %) of the families have only either one or two member(s). The highest number of members is observed in the miR171 family followed by miR477, miR169 and miR167 with 14, 10, 9 and 8 members, respectively. In the remaining 49 families, the number of miRNA varies from two to seven (Fig. 4). This distribution is in agreement with the diversity observed in other plant species [57]. Figure 5 shows the distribution of different miRNAs across the 11 chromosomes of *C. cajan*.

**Table 1:** Distribution of miRNAs across various miRNA families.

Number of members	miRNA families	Number of miRNA families
1	miR403, miR417, miR444, miR476, miR478, miR535, miR771, miR774, miR781, miR816, miR825, miR827, miR831, miR835, miR838, miR854, miR857, miR900, miR952, miR1025, miR1039, miR1061, miR1087, miR1088, miR1097, miR1128, miR1130, miR1153, miR1171, miR1217, miR1426, miR1428, miR1430, miR1439, miR1446, miR1507, miR1510, miR1518, miR1520, miR1535, miR1852, miR1854, miR1916, miR1917, miR2055, miR2079, miR2086, miR2090, miR2101, miR2102, miR2105, miR2108, miR2118, miR2119, miR2199, miR2275, miR2600, miR2604, miR2608, miR2611, miR2642, miR2646, miR2657, miR2671, miR2866, miR2871, miR2878, miR2905, miR2912, miR2920, miR2923, miR2928, miR3433, miR3436, miR3438, miR3441, miR3444, miR3447, miR3512, miR3515, miR3522, miR3626, miR3627, miR3629, miR3630, miR3631, miR3704, miR3712, miR3950, miR3979, miR4223, miR4237, miR4238, miR4244, miR4249, miR4340, miR4414, miR5039, miR5055, miR5075, miR5139, miR5140, miR5171, miR5174, miR5183, miR5201, miR5205, miR5219, miR5234, miR5237, miR5248, miR5253, miR5261, miR5264, miR5265, miR5285, miR5288, miR5291, miR5292, miR5368, miR5372, miR5373, miR5374, miR5379, miR5382, miR5521, miR5523, miR5532, miR5555, miR5558, miR5561, miR5668, miR5672, miR5716, miR5722, miR5745, miR5757, miR5770, miR5773, miR5775, miR5778, miR5828, miR5837, miR6034, miR6111, miR6140, miR6148, miR6173, miR6182, miR6191, miR6196, miR6230, miR6231, miR6271, miR6291, miR6443, miR6449, miR6457, miR6459, miR6462, miR6466, miR6476, miR6478, miR6483, miR6485, miR7124, miR7125, miR7127, miR7484, miR7488, miR7508, miR7516, miR7532, miR7534, miR7540, miR7545, miR7696, miR7728, miR7736, miR7741, miR7745, miR7753, miR7757, miR7767, miR7812, miR7814, miR7816, miR7817, miR7982, miR8007, miR8014, miR8030, miR8035, miR8044, miR8047, miR8049, miR8123	197
2	miR158, miR160, miR161, miR162, miR164, miR168, miR394, miR397, miR398, miR408, miR414, miR419, miR530, miR837, miR846, miR862, miR868, miR1023, miR1027, miR1030, miR1046, miR1051, miR1134, miR1320, miR1508, miR1511, miR1512, miR1514, miR1525, miR1527, miR1533, miR1534, miR2089, miR2093, miR2595, miR2606, miR2607, miR2628, miR2630, miR2641, miR2650, miR2655, miR2665, miR2673, miR2868, miR3434, miR3711, miR3951, miR4233, miR4246, miR4248, miR4371, miR4376, miR4413, miR4415, miR5040, miR5041, miR5054, miR5057, miR5142, miR5240, miR5255, miR5256, miR5257, miR5260, miR5281, miR5369, miR5512, miR5559, miR5565, miR5712, miR5721, miR5741, miR6135, miR6169, miR6202, miR6218, miR6232, miR6281, miR6299, miR6300, miR6464, miR7535, miR7543, miR7742, miR7776, miR7822, miR7823, miR7834, miR8051, miR8140	91
3	miR390, miR395, miR400, miR437, miR828, miR829, miR859, miR860, miR902, miR1044, miR1438, miR1521, miR1863, miR2111, miR2676, miR2873, miR2931, miR4245, miR5163, miR6025, miR6470, miR7699, miR8011, miR8041	24
4	miR159, miR172, miR393, miR399, miR1516, miR2592, miR3513, miR5031, miR7701, miR8005, miR8040	11
5	miR166, miR821, miR1078, miR1435, miR1522, miR5185, miR6288	7
6	miR319, miR396, miR1530, miR5568	4
7	miR156, miR482, miR845	3
8	miR167	1
9	miR169	1
10	miR477	1
14	miR1710	1

**Fig. 6:**
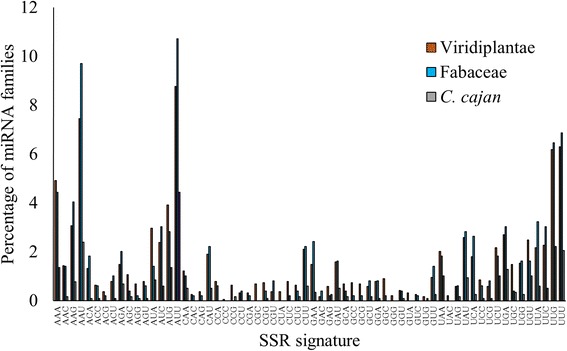
Distribution of SSR signatures in Viridiplantae, Fabaceae and *C. cajan*

The SSR signatures in various miRNA families of the kingdom Viridiplantae, the family Fabaceae and the species *C. cajan* are presented in Table 2, while their relative distributions are shown in Fig. 6. We observe only 45 signature SSRs present in the miRNA families of *C. cajan*. The highest frequency is observed for AAU in Viridiplantae, Fabaceae and *C. cajan*. A total of 19 SSRs are absent in miRNA families of *C. cajan* while 11 of them are present only in one family. The signatures GUG and CCG are absent in other Fabaceae species while they are present in *C. cajan*. The former signature is present in miR1171 while the latter is present in miR2102 and miR5075 families.

**Table 2:** Distribution of SSR signatures in various miRNA families of Viridiplantae, Fabaceae and *C. cajan*

	A			C			G			U			
	V^a^	F^b^	C^c^	V^a^	F^b^	C^c^	V^a^	F^b^	C^c^	V^a^	F^b^	C^c^	
A	4.92	4.44	1.36	1.32	1.82	0.09	1.48	2.02	0.68	2.96	1.41	0.85	A
	1.43	1.41	0.17	0.63	0.61	0.09	1.06	0.40	0.17	2.38	3.03	0.60	C
	3.07	4.04	0.77	0.37	0.20	0.00	0.69	0.20	0.09	3.91	2.83	1.36	G
	7.45	9.70	2.39	0.79	1.01	0.09	0.79	0.61	0.09	8.77	10.71	4.43	U
C	1.22	1.01	0.51	0.79	0.61	0.00	0.32	0.20	0.00	0.37	0.00	0.00	A
	0.26	0.20	0.00	0.05	0.00	0.00	0.69	0.00	0.00	0.79	0.20	0.00	C
	0.37	0.20	0.00	0.63	0.00	0.17	0.74	0.40	0.09	0.63	0.40	0.17	G
	1.90	2.22	0.51	0.32	0.40	0.00	0.37	0.81	0.00	2.11	2.22	0.60	U
G	1.48	2.42	0.34	0.69	0.40	0.17	0.79	0.81	0.09	0.32	0.00	0.00	A
	0.16	0.40	0.00	0.74	0.20	0.00	0.90	0.20	0.00	0.26	0.20	0.00	C
	0.58	0.20	0.26	0.69	0.20	0.00	0.21	0.00	0.00	0.16	0.00	0.09	G
	1.59	1.62	0.51	0.58	0.81	0.17	0.42	0.40	0.09	0.95	1.41	0.26	U
U	2.01	1.82	1.02	1.80	2.63	0.26	2.70	3.03	1.28	2.17	3.23	0.60	A
	0.21	0.00	0.00	0.85	0.61	0.09	1.48	0.40	0.34	2.27	3.03	0.51	C
	0.58	0.61	0.17	0.58	0.81	0.09	1.53	1.62	0.26	6.18	6.46	2.22	G
	2.59	2.83	0.94	2.17	1.82	1.02	2.48	1.62	1.02	6.29	6.87	2.05	U

V^a^- The percentage of miRNA families belonging to Viridiplantae with a particular signature SSR

F^b^- The percentage of miRNA families belonging to Fabaceae with a particular signature SSR. The data for V^a^ and F^b^ are taken from our previous study [16] C^c^- The percentage of miRNA families belonging to *C. cajan* with a particular signature SSR. There are 118 miRNA families to which *C. cajan* miRNAs belong

### miRNA targets on coding sequences

The mature miRNAs play a major role in the regulation of gene expression either by inhibiting translation or by degrading coding mRNAs [58, 59]. The number of targets for an miRNA may range from one to hundreds [60]. However, many mRNA targets in plants contain single miRNA-complementary site, which perfectly complement with the corresponding miRNAs and cleave the target [61]. We have used the psRNATarget server for the prediction of miRNA targets. Due to the absence of *C. cajan* target candidates in the psRNATarget server, the CDS sequences of *C. cajan* were used as target candidates. For 259 miRNAs, belonging to 180 families, 1373 target sequences were predicted. In order to characterise the targets, BLASTX was used with the predicted target sequences as query and the entire protein sequences of Viridiplantae as subject. Using 80% sequence identity cut-off, 298 targets for 122 miRNAs were characterised (Additional file 2: Table S2).

In majority of the cases, the predicted targets in this study were in accordance with the already published reports in other plant species. Wu, et al. [62] have showed that miR156 and miR172 families work in coordination to regulate the transition from the juvenile to the adult phase of plants. miR156 targets squamosa promoter binding protein-Like (SPL) transcription factor (TF) gene family to control the transition from the vegetative phase to the floral phase in *Arabidopsis*, rice and maize [63–68]. The cca-miR156b also targets SPL and is in agreement with the observation found in the literature. Members of the miR164 family target the NAC family of TF genes in *A. thaliana*, *Picea abies* and *Vitis vinifera *[69–73]. The NAC family of TFs play a major role in regulation of the boundary domain around developing primordia at the shoot apical and floral meristems [74]. cca-miR164e also targets NAC domain proteins. Scarecrow-like transcription factor is already an established target for miR171 family in *Arabidopsis *[75] and *Oryza sativa* [76]. Similar results were obtained in our study where cca*-*miR171b was predicted to bind Scarecrow-like (SCL) TF. SCL TFs are known to negatively regulate chlorophyll biosynthesis by suppressing the expression of the key gene PROTOCHLOROPHYLLIDE OXIDOREDUCTASE (POR). The miR172 family control plant development by regulating the trichome growth in *Arabidopsis *[62]. It is already established that MYB transcription factors are the negative controllers of the trichome growth [77]. The cca-miR172b family targets the MYB transcription factor mRNAs, and by cleaving these transcription factors they positively control the trichome growth. miR172 functions in regulating the transitions between developmental stages and in specifying floral organ identity. During flower development, miRNA172 represses the expression of APETALA2 (AP2) [78]. This regulation is crucial for the proper development of the reproductive organs and for the timely termination of floral stem cells [79]. The cca-miR172c targets floral homeotic protein AP2. The cca-miR397a targets laccase (LAC) enzymes, and is in agreement with established targets of miR397 family in *A. thaliana*, *Populus trichocarpa* and *O. sativa*. In rice, it is reported that the miR397 overexpression leads to greater number of branches, increased number of grains per main panicle, increased grain size and substantially enhanced grain yield. In case of *A. thaliana*, overexpression of miR397b causes a reduction in lignification of vascular and interfascicular tissue as well as an increase in inflorescence shoots number and seed size.

**Fig. 7:**
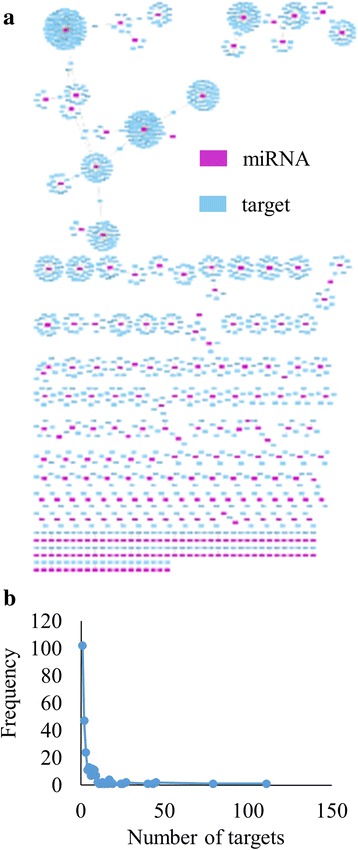
miRNA targets on coding sequences. **a** Interaction network of miRNA and protein coding targets. **b** Frequency distribution of number of miRNA targets.

The UniProt proteome, UP000075243, was used to map the target mRNAs and retrieve the corresponding UniProt protein identifiers. Of the 1373 targets, 1312 were mapped to the proteome. The visualization of these targets is provided as an interaction network between the miRNA and the corresponding UniProt entry in Fig. 7(a). The network consists of 1525 nodes. The number of targets ranges from 1 to 111 mRNAs (Fig. 7(b)). The highest number of targets is observed in cca-miR8123a. The characterized targets for cca-miR8123a includes mRNAs coding for ribosomal proteins, chaperones, kinases, transporters, receptors, signal transducers, ubiquitination proteins and spliceosomal RNAs (Additional file 2 Table S2). Another major node in the interaction network is cca-miR902a with 79 targets. The targets include mRNAs coding for RNA polymerases, kinases, U-box proteins, methyltransferase, retrotransposon proteins, hydrolases, kinases and CLIP-associated proteins. There are 102 miRNAs which target only one mRNA. For example, cca-miR171k targets the mRNA, which codes for F-box protein.

**Fig. 8:**
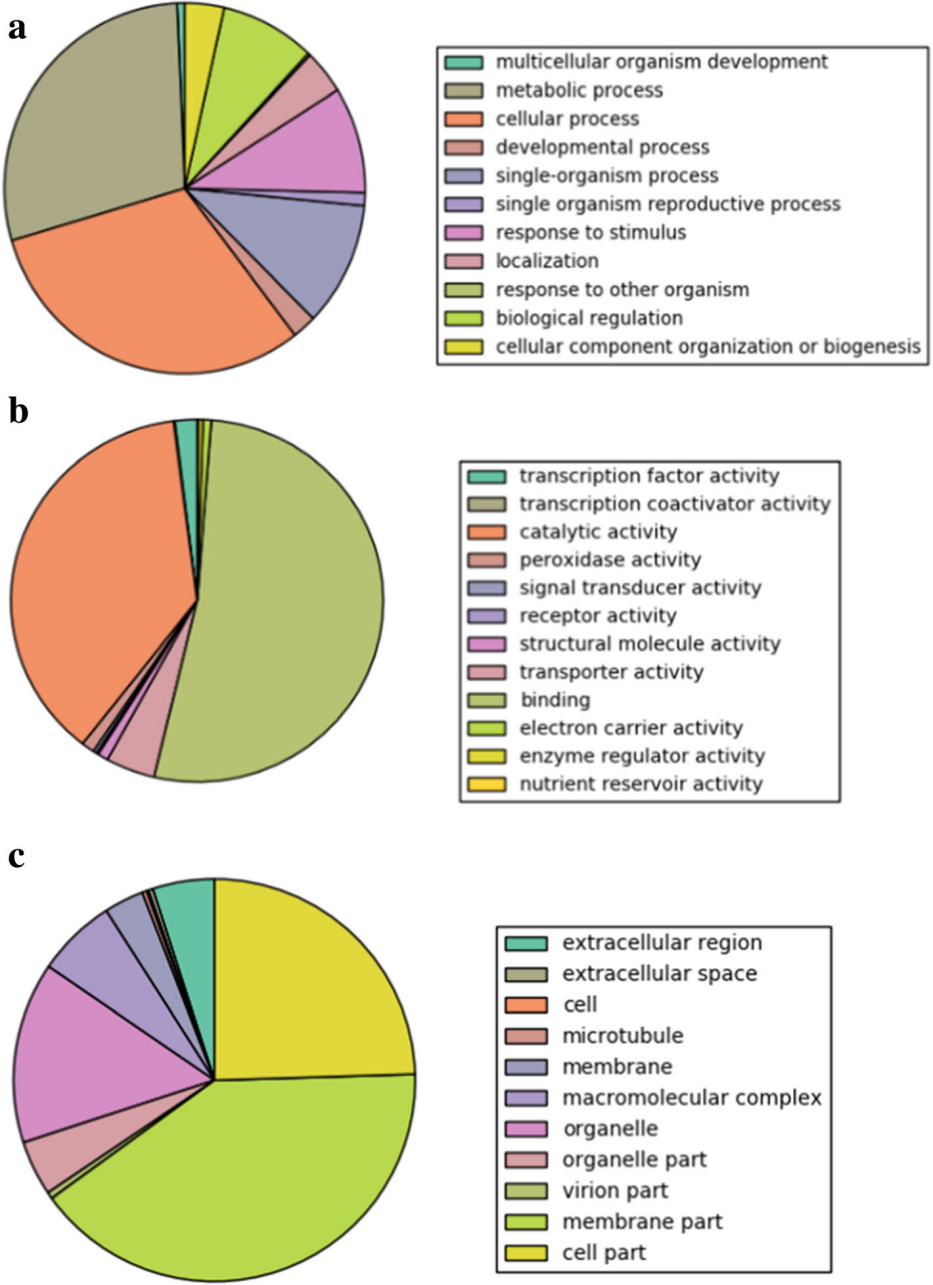
Targets of miRNA distributed among three different GO terms: (**a**) Biological processes, (**b**) Molecular functions and (**c**) Cellular components

The GO annotations for the targets were taken from UniProt-GOA. The biological processes, molecular functions and cellular components of the targets are shown in Fig. 8. Under Biological process, majority of the targets (60 %) are involved in the metabolic and cellular process (Fig. 8(a)). Around 10 % of the targets are responsible for the response to stimuli, while 8.5 % are involved in the regulation of biological process. The remaining (21.5 %) are involved in a plethora of processes including reproduction, development, component organization, localization and other cellular processes. The molecular functions performed by the targets cover almost all aspects of plant metabolism (Fig. 8(b)). Majority of the targets perform functions in binding (52.5 %) and catalytic activity (37.5 %). The remaining 10 % functions in nutrient reservation, transportation, signal reception and transduction, transportation and regulatory activities. The proteins coded by miRNA targets localize in different cellular components (Fig. 8(c)). A large number of proteins localize in membrane and membrane parts (43.7 %), protoplasm (24.5 %), cell organelles (19.0 %), macromolecular complexes (6.4 %) and extracellular region (5.3 %). The remaining (1.1 %) are localized in microtubules, virion parts and other regions in cell.

### Prediction of lncRNAs

The full length cDNA sequences of *C. cajan* were used as the starting point for predicting the lncRNAs. The sequences longer than 200 nucleotides and does not have an ORF coding for more than 120 residues were only selected as the input for prediction pipeline. The coding potential of these sequences were used as a measure to remove the potential protein coding sequences and to retain the non-coding sequences. A total of 3919 lncRNAs were predicted by this pipeline.

lncRNAs have emerged as important regulators of gene expression in a variety of biological processes in multiple species. lncRNAs are increasingly recognized as functional regulatory components in eukaryotic gene regulation. In plants, they are transcribed by different RNA polymerases and show diverse structural features. Recent studies have showed that the lncRNAs play a major role in growth and cell differentiation [80], phosphate homeostasis [81], chromatin modification [82, 83] and protein re-localization [84, 85]. Three major mechanisms of action are mainly proposed for the functioning of lncRNAs: decoys, scaffolds and guides [86]. lncRNAs act as decoys that prohibit the access of regulatory proteins to DNA. They also act as adaptors to bring two or more proteins into discrete complexes and guides in localizing specific proteins [87]. The miRNA target mimicking by lncRNA can be exemplified with *Induced by Phosphate Starvation1* (*IPS1*) lncRNA, which has a stretch of 23 conserved nucleotides that is partially complementary to miR399. The *IPS1* acts as a non-cleavable target mimic for miR399 in *Medicago truncatula* [88], rice [89] and *Arabidopsis* [90, 91]. Chromatin remodelling is demonstrated by the action of two classes of lncRNAs identified in the regulation of *FLC* (*Flowering Locus C*) expression. *FLC* is a floral repressor, which is repressed during the process of vernalization and it is mediated by polycomb repressive complex *PRC2*, which is a repressive chromatin modifier. Two classes of lncRNAs – cold induced antisense intragenic RNA (*COOLAIR*) and cold assisted intronic non-coding RNA (*COLDAIR*) are involved in this process of stable silencing of FLC [82, 92–94]. The transcription of *COOLAIR* is repressed in warm temperatures by stabilization of a RNA-DNA hybrid structure (R-loop) in its promoter region [95]. The COLDAIR is involved in the enrichment of H3K27me3 by direct interaction with CURLY LEAF (CLF), which is a component of *PRC2*, thereby repressing *FLC* [82].

### Prediction of miRNA targets on lncRNAs

**Fig. 9:**
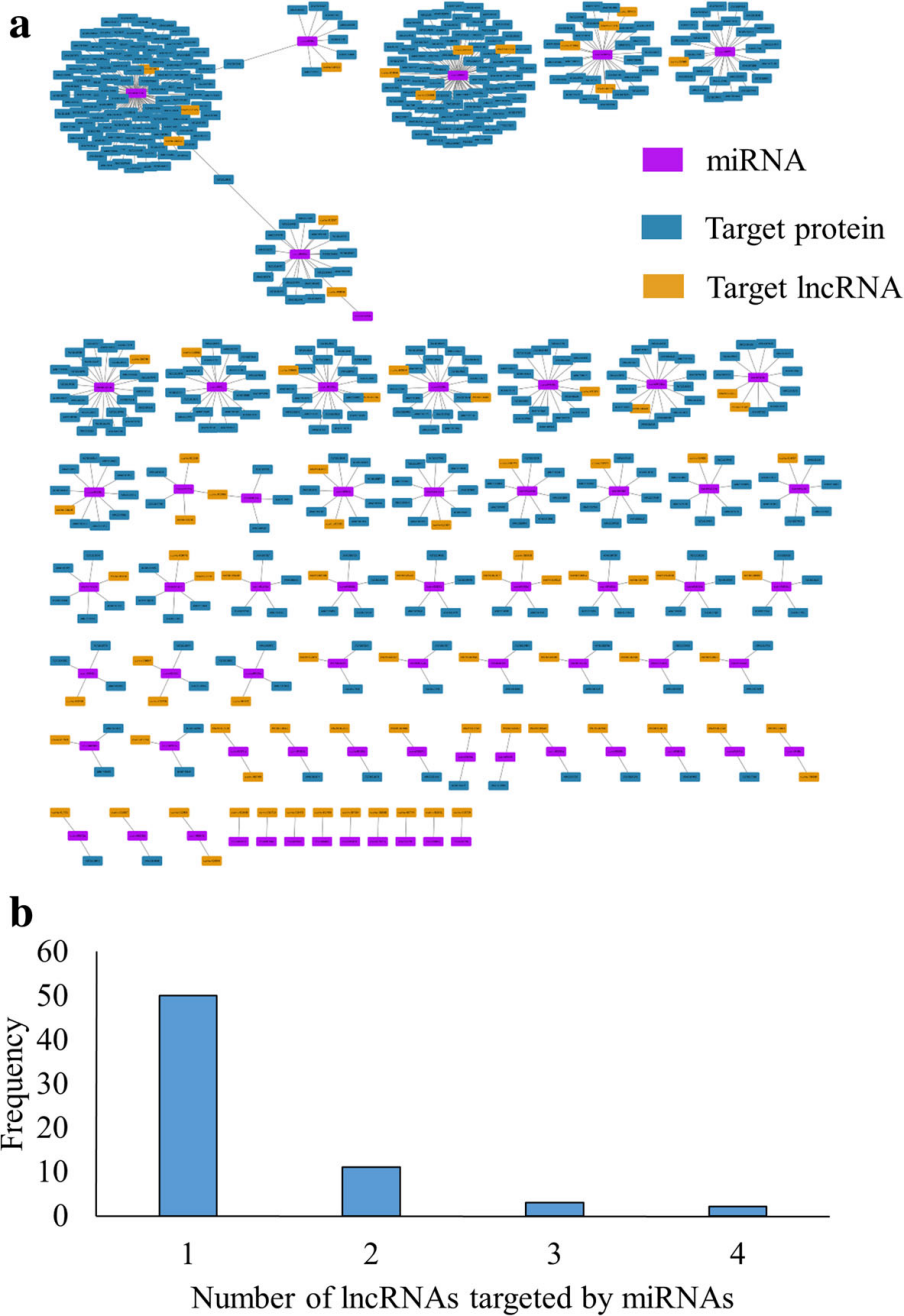
miRNA targets on lncRNAs. **a** Interaction network of miRNA and their coding and non-coding targets. (**b**) Frequency distribution of number of miRNA targets on lncRNAs

In this study, we have identified both the miRNAs and lncRNAs belonging to *C. cajan*. In order to study the direct targeting of lncRNAs by miRNAs, we have identified the targets of lncRNAs on miRNAs using psRNATarget server. A total of 66 miRNAs were identified to target 87 lncRNAs. The details of miRNAs, targeting lncRNAs, are available in the Additional file 3: Table S3. The interaction network of miRNAs that target lncRNAs is shown in Fig. 9(a). The network consists of 665 nodes. The number of lncRNAs targeted by a single miRNA varies from one to four, with a majority of them (76 %) targeting only one (Fig. 9(b)). cca-miR3979a and cca-miR902a targets four lncRNAs. These miRNAs also have relatively higher number of protein targets, 26 and 79 respectively. cca-miR8123a, which has the highest number of proteins targets, has three lncRNAs as targets. cca-miR1527a and cca-miR403a has three target lncRNAs, however, both of them target two proteins each.

## Conclusion

In the present study, we have identified the miRNAs from the genome of *C. cajan* and their corresponding targets. A total of 616 miRNAs belonging to 341 different families were identified. Of the identified miRNAs, 578 are novel that are not reported in the MiRBase 21. We have also identified 1379 targets for 259 miRNAs, of which 298 were characterized at protein level. Moreover, we have identified 3919 lncRNAs of *C. cajan,* 87 of which are found to be targeted by 66 miRNAs. It is well known that ncRNAs and their target mimics has the potential to be used for crop improvement programmes as proper management of them can generate crop cultivars with improved agronomic traits leading to increased yield and high nutritional value. Thorough understanding of interaction of miRNAs and their targets can provide valuable insight into molecular pathways controlling plant stress responses. Accordingly, our findings will enhance the knowledge of ncRNAs in economically important pulse crop *C. cajan* and their role in PTGR, will contribute in genome editing and thereby development of better crop varieties.

## Additional files

Additional file 1: Table S1. Predicted miRNAs of C. cajan. (PDF 955 kb)

Additional file 2: Table S2. Predicted targets of C. cajan miRNAs. (PDF 193 kb)

Additional file 3: Table S3. miRNAs targeting lncRNAs in *C. cajan.* (PDF 46 kb)

## Abbreviations

PTGR: post transcriptional regulation of gene expression
ncRNAs: non-coding RNAs
sncRNAs: small non-coding RNAs
lncRNAs: long non-coding RNAs
miRNAs: microRNAs
ceRNAs: competing endogenous RNAs
pre-miRs: precursor miRNAs
MFEI: Minimal Folding Free Energy Index
NQ: normalised Shannon entropy
Npb: normalized base-pairing propensity
ND: normalized base-pair distance
SSR: simple sequence repeat
CDS: coding DNA sequences
ORF: open reading frame
