# Supplementary material for: Genome-wide identification of miRNAs and lncRNAs in Cajanus cajan
Source: BMC Genomics. 2017 Nov 15;18:878. doi: 10.1186/s12864-017-4232-2 (PMC5688659; doi:10.1186/s12864-017-4232-2)
Supplement: Supplementary file 1 — Predicted miRNAs of C. cajan. (PDF 955 kb) [file 12864_2017_4232_MOESM1_ESM.pdf]

Table S1 - Predicted miRNAs of *C. cajan*.

Family	Member miRNA(s)	Mature Sequence	SSR[a]	LM[b]	LP[c]	AU[d]	MFEI[e]	Npb[f]	NQ[g]	ND[h]	R[i]
	cca-miR156a	GGUGUCAGAGAGAGAAUUGAG		21	158	70.89	0.49	0.27	0.43	0.14	3.80
	cca-miR156b	CAGAAGAGAGAGAGCACAA		19	435	57.93	0.58	0.31	0.54	0.16	3.45
	cca-miR156c	UAUGAUAGAAAGAGAGAGAGC		21	239	66.95	0.53	0.27	0.54	0.16	4.60
	cca-miR156d	UGAUGACAGAAGUAUAGAGAGC		22	126	61.90	0.96	0.35	0.15	0.05	3.17
	cca-miR156e	UGUGCUUUCUAUCUUCUGUCAA		22	108	62.04	1.01	0.32	0.10	0.04	2.78
	cca-miR156f	GCUGACAGAUAGAGAAGUGAGC		22	132	53.03	0.94	0.35	0.07	0.02	3.03
156	cca-miR156g	GCUCACUUCUCUAUCUGUCAGC	AGA	22	103	49.51	1.18	0.41	0.04	0.02	2.91
	cca-miR158a	CUUUGUCCACAAUUUUGGAA		20	60	76.67	0.41	0.33	0.44	0.15	5.00
158	cca-miR158b	CCAAAAUUGUAGACAAA	UGU	17	91	63.74	0.42	0.31	0.26	0.09	6.59
	cca-miR159a	GAGCUCCUUGAAGUCCAAA		19	109	55.96	0.74	0.30	0.14	0.05	3.67
	cca-miR159b	UUGGAUCGAAUGGAGCUCCA		20	91	56.04	0.52	0.34	0.28	0.09	3.30
	cca-miR159c	UUUGGUUUGAAGGGGGCUCUG		21	405	58.77	0.61	0.31	0.37	0.12	3.95
159	cca-miR159d	AACAGAUGCUGCAGCAGCUG	UUG	20	357	49.86	0.68	0.31	0.40	0.12	4.48
	cca-miR160a	AAUGGCAUACAGGGAGCCAGGCA		23	105	50.48	1.08	0.36	0.02	0.01	5.71
160	cca-miR160b	UGCCUGGCUCCCUGUAUGCCAUU	AUG	23	113	53.98	1.11	0.38	0.05	0.02	6.19
	cca-miR161a	UAGUCACUUUCAAUUCAUU		19	139	63.31	0.67	0.30	0.34	0.11	4.32
161	cca-miR161b	UCAAUACAUUGAAACUGACU	UUU	20	143	75.52	0.59	0.27	0.30	0.09	2.80
	cca-miR162c	UGGAGGUAGCGGUUCAUCGAUC		22	504	70.83	0.58	0.26	0.42	0.12	3.37
162	cca-miR162d	UCGAUAAACCUCUGCAUCCAG	AUC	21	131	58.02	0.83	0.36	0.10	0.04	3.82
	cca-miR164e	UGGAGAAGCAGGGCACUUGCU		21	119	50.42	0.60	0.32	0.24	0.08	3.36
164	cca-miR164f	UGCACGUGCUCUCCUUUUCCA	GCA	21	112	58.04	0.79	0.27	0.04	0.01	3.57
	cca-miR166g	GGGAAUGAAGCCUGGUCCGA		20	119	49.58	0.84	0.34	0.14	0.05	2.52
	cca-miR166h	UCGGACCAGGCUUCAUUCCCC		21	234	58.97	0.52	0.28	0.28	0.08	6.41
	cca-miR166i	CCUCGAGCCAGACAACAUUCC		21	116	52.59	0.84	0.35	0.12	0.05	2.59
	cca-miR166j	GGAAUGUUGUUUGGCUCGAGG		21	228	65.79	0.90	0.37	0.07	0.03	3.95
166	cca-miR166k	AUCUUGGAUCAUCCUUCAUU	UUC	20	412	60.92	0.70	0.30	0.29	0.09	2.91

	cca-miR167a	AAAUGAUGCUGUCAUUCAUGAU		22	260	62.69	0.71	0.32	0.34	0.12	5.00
	cca-miR167b	AUGAGGAUGUUAUGCAUGAUC		21	190	75.79	0.94	0.35	0.37	0.11	5.79
	cca-miR167c	GAUCAUGUUCGGGCUUCAC		19	76	51.32	0.62	0.30	0.11	0.05	2.63
	cca-miR167d	GAUCUUGCUGUAGCAGCCUCAC		22	473	62.58	0.65	0.33	0.44	0.14	2.54
	cca-miR167e	GGUGAAGCAAGCAUAUGAU		19	478	60.67	0.62	0.30	0.38	0.12	3.35
	cca-miR167f	CAGUUGAAGCUGCCAGCAUGAU		22	83	56.63	1.23	0.37	0.02	0.01	4.82
	cca-miR167g	AUCAUGUGGGAGUUUCAUU		19	287	63.41	0.59	0.31	0.42	0.14	2.79
167	cca-miR167h	UAAGAUCAUGCUGGCAGCUUCA	UGA	22	136	68.38	1.04	0.36	0.06	0.03	3.68
	cca-miR168a	GAUCCCGCCUUGCAUCAACUGAAU		24	127	40.94	0.87	0.40	0.10	0.04	4.72
168	cca-miR168b	UCGCUUGGUGCAGGUCGGGA	CGG	20	131	41.98	0.86	0.39	0.09	0.04	4.58
	cca-miR169a	GGCAAGUUUCUCUUGGCUACA		21	113	57.52	1.11	0.38	0.06	0.02	7.08
	cca-miR169b	CAGCCAAGGGUGAUUUGCCGG		21	124	57.26	1.10	0.38	0.06	0.02	6.45
	cca-miR169c	UGAGCCGGGAUGGCUUGCCGGCA		23	109	51.38	1.10	0.39	0.16	0.06	3.67
	cca-miR169d	CCGGCAAGUCAUUCUUGGCUC		21	72	59.72	1.08	0.29	0.07	0.03	2.78
	cca-miR169e	CAGGCAAAUCAUCCUUGGCUU		21	471	63.91	0.65	0.30	0.40	0.12	4.25
	cca-miR169f	UGAGCCAAGGAUGAGUUGCCU		21	492	55.08	0.59	0.30	0.36	0.11	2.64
	cca-miR169g	UAGCCAAGGGGACUGCACAUG		21	181	64.09	0.81	0.33	0.18	0.07	3.31
	cca-miR169h	AGCCAAUAAUGGCUUGCCU		19	120	40.83	0.78	0.38	0.13	0.05	2.50
169	cca-miR169i	UAGCCAUGUAUGAUUUGCCUG	UUG	21	497	61.97	0.53	0.29	0.51	0.15	3.22
	cca-miR171a	UCUGAUUGAACCGCACCAAUA		21	100	60.00	0.75	0.35	0.04	0.02	3.00
	cca-miR171b	UGAUUGAGCCGUGCCAAUAUC		21	114	57.89	0.88	0.32	0.13	0.04	3.51
	cca-miR171c	GAUAUUGGCACGGCUCAAUCA		21	468	66.88	0.61	0.31	0.40	0.13	2.99
	cca-miR171d	UAUUGGUCCGGUUCAAUGAGA		21	79	54.43	0.91	0.34	0.06	0.02	2.53
	cca-miR171e	UGAGCCGCUCCAAUAUCAC		19	500	46.20	0.65	0.34	0.45	0.15	4.00
	cca-miR171f	AGAUAUUGAAGUUGCUCAAUC		21	122	65.57	0.69	0.33	0.09	0.03	4.92
	cca-miR171g	CGAGCCGAAUCAAUAUCACUC		21	98	59.18	0.78	0.32	0.04	0.02	4.08
	cca-miR171h	GAGUAAUAUUGGUUCGGCUCG		21	104	60.58	0.88	0.37	0.18	0.07	2.88
	cca-miR171i	CGUGAUAUUGGUCCGGCUCAU		21	102	59.80	1.03	0.37	0.05	0.02	3.92
	cca-miR171j	UUAUUGAAUCGGACCAAUAUC		21	97	56.70	0.51	0.32	0.10	0.04	5.15
	cca-miR171k	UUUGAUGGAACCAUGCCAA		19	383	68.41	0.75	0.35	0.52	0.15	4.18
171	cca-miR171l	AUAAGAAAGCAGUGAUCAAA	UUG	20	363	72.73	0.71	0.31	0.23	0.07	2.75

	cca-miR171m	UGAGAUAUUGACGCGGCUCAA		21	113	57.52	0.84	0.35	0.08	0.03	3.54
	cca-miR171n	UUGGUGAGGUUCAAUCCGA		19	315	63.17	0.79	0.33	0.14	0.05	2.86
	cca-miR172a	AUGUAGCAUCAUCAAGAUUCA		21	160	59.38	0.98	0.36	0.18	0.06	5.00
	cca-miR172b	UGAAUAUUGAAGAUGCU		17	465	40.86	0.81	0.36	0.44	0.15	2.80
	cca-miR172c	GGAAUCUUGAUGAUGCUGCAGCAG		24	128	57.81	1.09	0.39	0.08	0.04	3.91
172	cca-miR172d	UGUGAAUCUUGAUGCUGCU	AUG	19	175	60.00	0.97	0.33	0.14	0.04	2.86
	cca-miR319a	UUGGUCUGAAGGGUUCCCU		19	73	49.32	0.47	0.33	0.24	0.07	2.74
	cca-miR319b	AGAGCUUUCUUCAGUCCACUC		21	494	65.38	0.64	0.34	0.27	0.09	3.44
	cca-miR319c	GGAGUUCCUUGCAGCCCAAAG		21	110	56.36	1.04	0.38	0.03	0.01	6.36
	cca-miR319d	GGGAGCUCCCUUCAGUCCAAG		21	194	61.34	0.87	0.30	0.07	0.02	2.58
	cca-miR319e	UGGACUGAAGGGGAGCUCCUUC		22	82	46.34	0.96	0.39	0.12	0.05	7.32
319	cca-miR319f	GGAUGAAUGAGUCGGCAGC	CUU	19	179	58.10	0.87	0.32	0.07	0.03	3.35
	cca-miR390a	UGAAACUCAGGAUAGAUAGCG		21	90	53.33	1.12	0.37	0.01	0.01	3.33
	cca-miR390b	AAGCUCAGGAGGGAUAGCACC		21	103	57.28	1.00	0.39	0.05	0.02	3.88
390	cca-miR390c	GGCGCUAUCUAUCCUGAGCUU	AUC	21	120	58.33	0.94	0.35	0.05	0.02	4.17
	cca-miR393a	AAGGGAUAGCAUGAUCCCAAA		21	110	59.09	1.10	0.34	0.14	0.05	3.64
	cca-miR393b	UUUGGGAUCAUGCUAUCCCUU		21	100	60.00	1.21	0.35	0.08	0.03	6.00
	cca-miR393c	GGAUCAAUGCGAUCCCUUUGGA		22	113	58.41	1.06	0.33	0.14	0.05	3.54
393	cca-miR393d	UCCAAAGGGAUCGCAUUGAUCC	AUC	22	115	57.39	1.16	0.36	0.07	0.02	5.22
	cca-miR394a	AGGUGGGCAUACUGCCAACU		20	475	61.05	0.61	0.27	0.26	0.08	4.84
394	cca-miR394b	UUGGCAUUCUGUCCACCUCCAU	UCU	22	157	53.50	0.89	0.34	0.08	0.03	3.82
	cca-miR395a	GGAGUUCCUCCAAACACUUCA		21	114	60.53	0.86	0.32	0.17	0.06	2.63
	cca-miR395b	CUGAAGUGUUUGGGGGAACUCC		22	119	59.66	1.02	0.34	0.08	0.03	4.20
395	cca-miR395c	CAAUGAAGCGUUAAGGGGAAC	ACU	21	79	54.43	0.69	0.28	0.08	0.03	2.53
	cca-miR396a	GUUCAAUAAAGCUGUGGGAAG		21	123	52.85	0.69	0.28	0.17	0.06	4.07
	cca-miR396b	CUUCCCACAACUUUAUUG		18	155	58.06	0.62	0.34	0.28	0.09	2.58
	cca-miR396c	UUCCACAGCUUUGUUGCACUU		21	406	66.75	0.51	0.27	0.35	0.11	2.96
	cca-miR396d	GCAUAAGAAGUUCAAGAA		18	97	64.95	1.18	0.38	0.09	0.03	3.09
	cca-miR396e	UCCACAGGCUUUCUUGAACGG		21	225	51.56	0.58	0.33	0.26	0.08	3.11
396	cca-miR396f	UCUCCCACAGCUUUCUUGAGC	CUU	21	107	52.34	0.91	0.32	0.14	0.05	3.74
397	cca-miR397a	AUUGAGUGCAGCAUUGAUGAC	UGA	21	461	67.68	0.52	0.27	0.46	0.13	3.47

	cca-miR397b	CAUCAACGCUGCACUCAAUGA		21	133	60.15	0.87	0.35	0.20	0.07	6.02
	cca-miR398a	GGAGUGAAUCUGAGAACACAAG		22	101	60.40	1.13	0.41	0.08	0.03	2.97
398	cca-miR398b	UUGUGUUCUCAGGUCACCCCU	UCA	21	114	58.77	1.08	0.40	0.09	0.04	2.63
	cca-miR399a	GGGCUUCUCUUUCUUGGCAGG		21	104	59.62	1.08	0.33	0.10	0.04	3.85
	cca-miR399b	CUGCCAAUAGAGAAGUUGCCC		21	78	50.00	0.82	0.29	0.04	0.01	5.13
	cca-miR399c	CUGGGCAAAUCUCCUUUGGCA		21	113	53.98	0.78	0.29	0.10	0.03	6.19
399	cca-miR399d	UGCCAAAGGAGAUUUGCCCUG	UGC	21	115	54.78	1.18	0.40	0.04	0.01	5.22
	cca-miR400a	UUCAUGACAUUAUUAUAAG		19	56	73.21	0.86	0.34	0.04	0.02	3.57
	cca-miR400b	UCAUGAGAUUAUUCUAAGU		19	146	69.86	0.75	0.33	0.16	0.06	5.48
400	cca-miR400c	GACUUAUAAAAAUCUAAUGAA	AUU	21	505	74.85	0.60	0.28	0.37	0.11	5.74
403	cca-miR403a	GUUUGUGCGUGAAUCUAA	AUC	18	80	60.00	1.22	0.35	0.02	0.01	2.50
	cca-miR408a	CAGGGGAACAGGCAGAGCAUG		21	98	48.98	0.78	0.34	0.15	0.05	3.06
408	cca-miR408b	UGCACUGCCUCUUCCCUGGCU	AGA	21	110	50.00	0.85	0.35	0.11	0.03	2.73
	cca-miR414a	UCAUCGUCAUCAUCAUUGUCC		21	449	60.58	0.42	0.26	0.28	0.09	2.90
414	cca-miR414b	GACGAUGAUGAUAAGGAUG	CAU	19	416	71.88	0.77	0.31	0.23	0.07	3.13
417	cca-miR417a	GAAUAUAGUGAAUUAGUUCC	AAU	20	425	73.18	0.69	0.31	0.52	0.15	4.71
	cca-miR419a	AACAUCAUCAGCAUUCAUC		19	320	72.81	0.69	0.32	0.38	0.12	2.50
419	cca-miR419b	UGAUGAAUGAUGAUGAUGUAU	AUG	21	409	69.68	0.71	0.33	0.44	0.14	4.40
	cca-miR437a	AAGUUAUAGAACUCUGACUU		20	75	61.33	0.80	0.33	0.04	0.01	4.00
	cca-miR437b	AAAGGUAGAGAUGUUUGACU		20	388	65.72	0.65	0.27	0.30	0.09	3.09
437	cca-miR437c	AAAGUUUGAGAAGUUUGACU	UUU	20	436	66.51	0.64	0.28	0.34	0.10	3.44
444	cca-miR444a	UGUUGCCUCAAGUUUGCUGC	UGC	20	138	63.77	0.73	0.30	0.22	0.07	4.35
476	cca-miR476a	UAGUGAUCCUUCUUUUCAAA	AUU	20	501	65.87	0.68	0.31	0.31	0.10	3.39
	cca-miR477a	AGAGAAGCCAUUGAGAGAGA		20	141	55.32	0.51	0.28	0.30	0.11	2.84
	cca-miR477b	CUCUCCCUCAUGUUGUUCUA		20	400	70.75	0.61	0.26	0.30	0.09	2.50
	cca-miR477c	UAGAAGAACUUGAGAGAGAG		20	421	64.85	0.65	0.30	0.33	0.10	3.09
	cca-miR477d	CUCUUUUUGAAAGGCUUCU		19	83	68.67	0.72	0.37	0.21	0.08	7.23
	cca-miR477e	UAGAAGCAUUUGCAGAAGAG		20	221	75.57	1.26	0.32	0.20	0.07	2.71
	cca-miR477f	CUCUCCCUCAAGGGCUUCUC		20	119	52.10	0.86	0.34	0.07	0.03	2.52
	cca-miR477g	CUCUCUCUCAAAGGCCUCCA		20	135	51.85	0.74	0.33	0.12	0.04	2.96
477	cca-miR477h	GGAAGCCAUUGAAGGAGAU	CUU	19	499	66.73	0.64	0.31	0.30	0.09	2.61

	cca-miR477i	UGGAAGCCUUUGAGGGA		17	72	62.50	1.11	0.35	0.11	0.04	4.17
	cca-miR477j	AUCUCCUUCAAAGGUUUCCA		20	55	67.27	0.67	0.27	0.29	0.11	3.64
478	cca-miR478a	UAACGUGUACUUCUAUUUUUA	UUA	21	490	70.41	0.66	0.29	0.48	0.14	3.47
	cca-miR482a	AAUGGGAGGAACUAGGAAG		19	134	61.19	0.52	0.28	0.22	0.08	2.99
	cca-miR482b	CCUACUCGUCCCAUUCC		17	440	61.82	0.65	0.31	0.31	0.10	2.50
	cca-miR482c	UUGGAAUGGGACGUUUUGGAAA		22	150	54.00	1.09	0.37	0.03	0.01	3.33
	cca-miR482d	AGGAAUGGGCAGUUUAGGAAGA		22	477	66.46	0.69	0.33	0.33	0.10	2.73
	cca-miR482e	UUUCUUACUCCUCCCAUACC		20	149	57.05	0.56	0.32	0.17	0.06	3.36
	cca-miR482f	UUCCCUUUUCCUCCCAUU		18	274	54.74	0.62	0.32	0.44	0.13	4.01
482	cca-miR482g	GGAAUGGGUGGGAUAGGAAAGA	UUC	22	375	69.60	0.66	0.32	0.38	0.11	2.93
	cca-miR530a	UGCAUUUGCAGCUGCACUUU		20	91	58.24	1.04	0.40	0.09	0.04	8.79
530	cca-miR530b	GAAAGUGCAGGUGCAAGUGCA	UGC	21	145	51.03	0.86	0.28	0.14	0.04	6.21
535	cca-miR535a	AGUGACAACAAGAGAAAGCA	UGU	20	422	66.82	0.63	0.33	0.34	0.11	2.84
771	cca-miR771a	CAUGAAAAUCUGAGGAGCC	AUG	19	407	57.74	0.63	0.33	0.33	0.10	2.70
774	cca-miR774a	AGAUGAAGAUUGGGAUGAC	AUU	19	208	71.15	0.64	0.31	0.31	0.10	5.29
781	cca-miR781a	AAUUAGAGUUUUAAGGAUACU	UAA	21	255	76.86	0.79	0.30	0.18	0.06	5.88
816	cca-miR816a	UUGUAUUAAAAUAUGUCA	AUG	18	77	71.43	0.91	0.36	0.04	0.02	2.60
	cca-miR821a	AGUCCUCAACAACAAAGUU		19	270	63.70	0.60	0.29	0.39	0.12	3.70
	cca-miR821b	AAGUCAUCAAUAUUAAAGUUG		21	210	68.10	0.67	0.32	0.31	0.10	4.76
	cca-miR821c	CUUUUAUUUUGAUGACUU		18	433	67.44	0.56	0.30	0.41	0.12	4.16
	cca-miR821d	AAGUCUUCAAAAUAAAAGU		19	192	74.48	0.52	0.32	0.49	0.15	3.65
821	cca-miR821e	AAGUUAUCAAAACAAAAGUU	AAA	20	107	75.70	0.67	0.35	0.36	0.13	4.67
825	cca-miR825a	GCUUCUUCUAGUUGGUGCUUGA	AAG	22	305	58.36	0.58	0.31	0.33	0.11	3.28
827	cca-miR827a	GUAUGGAUGUCAAACAACAAA	CAU	21	300	72.00	0.62	0.31	0.25	0.08	3.67
	cca-miR828a	UUGCUUGCUCAAAUGAGCAUCU		22	435	73.56	0.62	0.29	0.39	0.12	3.22
	cca-miR828b	UAAUAAUUCUUGCUCUAAUGA		21	440	69.32	0.65	0.33	0.22	0.07	2.73
828	cca-miR828c	UGGAAUACUCAUUUGAGCAAGA	AUG	22	102	59.80	0.97	0.39	0.09	0.04	6.86
	cca-miR829a	UUUGAACCUUUGAUUUGAA		19	302	74.17	0.62	0.29	0.43	0.13	3.97
	cca-miR829b	ACUUUGAAUCAUUGAUUUG		19	341	71.55	0.71	0.32	0.35	0.12	2.64
829	cca-miR829c	UUCAAAUCAAAUGUUCAAAG	UGA	20	428	67.52	0.58	0.31	0.42	0.13	2.80
831	cca-miR831a	AGAAGAGGAAGAAGGAGAUGAGA	AAG	23	353	63.17	0.59	0.30	0.33	0.10	3.97

835	cca-miR835a	CUUUCUUGCUUUUCUUCUUCA	UCU	21	402	53.98	0.68	0.32	0.30	0.10	2.99
	cca-miR837a	ACGAAGAAGAAACUGAUGA		19	370	72.16	0.60	0.29	0.34	0.10	2.70
837	cca-miR837b	AUUGUUUUUUUUUUUUUCA	UUU	20	396	70.45	1.12	0.32	0.22	0.07	3.54
838	cca-miR838a	UUUUCUUCUUUUUCUUUCACA	UCU	21	487	75.98	0.89	0.31	0.39	0.11	3.08
	cca-miR845a	CAUCAUUUGGUAUUAGAGC		19	289	71.97	0.63	0.29	0.41	0.14	5.54
	cca-miR845b	GUUGAUCUGAUACCAAUUG		19	365	76.99	0.67	0.31	0.39	0.12	6.03
	cca-miR845c	AGCUAUGAUACCAAAUGAUA		20	316	72.15	0.59	0.31	0.35	0.11	4.75
	cca-miR845d	UAUCAAUUAGUAUCAAAGCU		20	270	61.11	0.68	0.34	0.40	0.13	3.33
	cca-miR845e	UAGCCCUGAUACCAAUUGA		19	262	67.56	0.50	0.26	0.47	0.13	2.67
	cca-miR845f	AGGCUCUGAUACCACUUGAUG		21	224	66.52	0.90	0.35	0.37	0.11	5.36
845	cca-miR845g	CAUCAAGUGGUAUCAGAGCCA	AUU	21	363	65.01	0.56	0.28	0.41	0.12	3.03
	cca-miR846a	AUUCAAUCACUUCAAUUCA		19	220	76.82	0.56	0.25	0.40	0.12	2.73
846	cca-miR846b	UUGACUUGAAAUGCUUGAAUU	UGA	21	407	61.92	0.64	0.33	0.34	0.10	2.95
854	cca-miR854a	AUGAUGAUAAUGAGGAGGAG	GAG	20	176	60.80	0.58	0.33	0.31	0.11	4.55
857	cca-miR857a	AAACUUACACCAUACAUAAUA	AAA	21	496	65.93	0.56	0.26	0.38	0.11	3.83
	cca-miR859a	UGAUUUUACAAAAGAUAGA		19	273	76.92	0.76	0.33	0.49	0.16	3.30
	cca-miR859b	AUUUUACAUUAGAUAGAUA		19	493	75.86	0.66	0.30	0.42	0.13	2.64
859	cca-miR859c	UAUCUAUCUAUUUUAAAAU	GAU	19	224	76.34	0.83	0.30	0.42	0.14	2.68
	cca-miR860a	UCAAUAGUUUGGACCAUAUAU		21	272	66.91	0.58	0.33	0.38	0.12	4.04
	cca-miR860b	UUCAAUAAAUUGGACUAUA		19	430	65.81	0.59	0.28	0.33	0.10	2.79
860	cca-miR860c	AUAUACUCCAAUGUAUUGAAG	AAU	21	361	65.37	0.63	0.29	0.34	0.11	3.05
	cca-miR862a	CUUCUAAGACAUCCAGC		17	431	63.57	0.68	0.32	0.42	0.13	3.25
862	cca-miR862b	UCCUUCAAGGACAUGCAGC	UUC	19	168	58.93	0.47	0.26	0.36	0.11	3.57
	cca-miR868a	UUCUUAAGUGUUGAUGAUGU		20	475	76.42	0.74	0.31	0.38	0.12	4.42
868	cca-miR868b	CAUUAUCAGUAGUUAAGAAG	AUA	20	174	64.37	0.48	0.26	0.50	0.14	4.02
900	cca-miR900a	UGUUCUUGUACCUGGGAA	AGA	18	152	63.16	0.68	0.30	0.29	0.10	2.63
	cca-miR902a	GAAGAAUCUGGAUCAU		16	232	65.95	0.74	0.33	0.33	0.11	3.88
	cca-miR902b	UUAUGAUGUUGAUCCUUCA		19	324	66.05	0.57	0.27	0.38	0.11	2.78
902	cca-miR902c	UUAUGUUGAAGAUUCUUCAU	GAU	20	102	58.82	0.53	0.30	0.43	0.14	5.88
952	cca-miR952a	AACUGAGAAUGAAAUUGGUG	AUU	20	467	72.38	0.68	0.30	0.35	0.11	4.93
1023	cca-miR1023a	GUAGGGAAAUGGAGAGUGUGU	UUG	21	373	44.50	0.57	0.33	0.32	0.11	3.75

	cca-miR1023b	ACUCUCUCGGUUUCCCUUC		19	356	60.96	0.64	0.33	0.41	0.12	2.81
1025	cca-miR1025a	UGCCACAACAAAGCAAAUAA	UGU	20	151	64.24	0.48	0.30	0.31	0.11	5.30
	cca-miR1027a	UUGGAAGAGAAGAAAGAAA		19	425	63.76	0.71	0.32	0.21	0.07	3.76
1027	cca-miR1027b	GAUUAGAAAAGAAGAUAGAA	AGA	20	499	76.95	0.74	0.31	0.43	0.11	2.81
	cca-miR1030a	UCUGCAUCUGCAUCUGCACC		20	378	40.48	0.61	0.32	0.30	0.10	3.17
1030	cca-miR1030b	UGGUGGAGGUGCAGAUGCA	UGC	19	75	48.00	0.70	0.31	0.17	0.06	4.00
1039	cca-miR1039a	UCUUUGUGUCUGUCUCUCCU	CUU	20	400	52.50	0.61	0.32	0.32	0.11	2.50
	cca-miR1044a	AAAACAAAUAUCCACUAC		18	489	61.76	0.69	0.33	0.41	0.13	2.66
	cca-miR1044b	UUGUAGUGCAUAGUUUGUUUU		21	91	67.03	0.55	0.33	0.29	0.11	6.59
1044	cca-miR1044c	GUUUUUAGUGCAUUUUUGUGC	UUU	21	62	69.35	0.95	0.32	0.11	0.04	4.84
	cca-miR1046a	GGUGAAAAAAAUGAAAAAU		19	497	74.04	0.62	0.28	0.46	0.13	2.62
1046	cca-miR1046b	UGGAUUGCAUAUUUUUCACG	UCA	20	499	65.73	0.53	0.28	0.42	0.13	2.81
	cca-miR1051a	UCUUCCUGUUUACUUGAA		18	242	73.55	1.06	0.31	0.26	0.08	4.13
1051	cca-miR1051b	GGUUCAAGUGAGAAGGAAGA	GAA	20	146	60.27	0.74	0.32	0.38	0.13	6.85
1061	cca-miR1061a	UGAGUAAUCUAUGAAAUAA	AUG	19	358	64.25	0.61	0.32	0.39	0.12	3.35
	cca-miR1078a	UUUGGAUAAUUAAAUUGUGAU		21	263	70.72	0.61	0.30	0.36	0.12	5.32
	cca-miR1078b	UUGGAUGAUUGAAAUGUGAU		20	155	74.19	0.86	0.35	0.11	0.04	7.10
	cca-miR1078c	AUCACAUUUUAAUCAUCCAA		20	451	73.84	0.74	0.27	0.26	0.07	4.88
	cca-miR1078d	AUCACAAUUAAAUAAAUCAA		20	480	74.17	0.59	0.32	0.39	0.12	4.58
1078	cca-miR1078e	UUGAUUGAAUCAGUUGUGAU	AUU	20	256	73.05	0.85	0.34	0.31	0.10	5.08
1087	cca-miR1087a	ACAGAUUGGUUUUAGUGCU	UUU	19	366	67.76	0.63	0.31	0.43	0.13	4.64
1088	cca-miR1088a	AGAAGAAAGAGUGCAGGCAU	UCU	20	271	66.42	0.50	0.29	0.41	0.12	2.58
1097	cca-miR1097a	GCCAAUGUUGUUGUUGGAA	UUG	19	349	57.88	0.54	0.29	0.38	0.11	4.58
1128	cca-miR1128a	UUUGGGACGGAGGGGGUAG	UAU	19	316	64.24	0.71	0.31	0.33	0.10	4.11
1130	cca-miR1130a	UCUUAUAUUAAGUGACAGA	UAA	19	334	71.56	0.73	0.32	0.45	0.12	2.99
	cca-miR1134a	CGACAACCACAGCAAGAAGAAGA		23	491	57.43	0.56	0.31	0.45	0.14	4.28
1134	cca-miR1134b	UCUUCUUGUUGUUGUUGUUGUUG	CAA	23	242	57.02	0.66	0.33	0.18	0.06	3.31
1153	cca-miR1153a	UUGUAGUUGCUAGCAUCACU	AUU	20	473	60.04	0.55	0.31	0.27	0.09	3.81
1171	cca-miR1171a	UGGAGUGGAGUGGAGUGGAGUGG	GUG	23	319	58.93	0.56	0.33	0.41	0.13	2.82
1217	cca-miR1217a	AAUUUGAAGGAUGUUGUCAAG	UUG	21	143	62.24	0.79	0.34	0.29	0.10	2.80
1320	cca-miR1320a	UUGGAACGAAUUUAUUUUAC	AUU	20	417	76.50	0.65	0.30	0.51	0.15	6.71

	cca-miR1320b	UAAAAUUCAUUCUUUCCAA		19	379	75.99	0.67	0.29	0.39	0.11	7.39
1426	cca-miR1426a	UUUAAUCAUAAACAAGAUUC	AAA	20	505	76.44	0.59	0.27	0.45	0.12	4.36
1428	cca-miR1428a	UAAGAUAAUGCUAUGAUUUCG	AUU	21	91	67.03	0.45	0.32	0.45	0.15	3.30
1430	cca-miR1430a	UGGUGAGCCUUCCUUGCUA	UGG	19	150	51.33	0.58	0.33	0.32	0.10	2.67
	cca-miR1435a	UUUCUUAAUUCAAACCUU		18	120	75.83	0.47	0.29	0.35	0.11	5.00
	cca-miR1435b	AAAAAGUUUAACUUAUGAAA		20	298	68.79	0.55	0.27	0.30	0.09	3.02
	cca-miR1435c	UUCUUCAGUCAAACUUUU		18	59	62.71	0.63	0.29	0.14	0.06	3.39
	cca-miR1435d	AAAAAAUUUGACUUAAGAAA		20	417	68.82	0.50	0.27	0.41	0.12	3.60
1435	cca-miR1435e	UUUCUUAAGUUAAAUUUUUU	AAA	20	496	76.01	0.69	0.32	0.45	0.14	3.63
	cca-miR1438f	AGGGUAAUUUGAUAAUUUUUA		21	334	75.45	0.90	0.30	0.34	0.09	5.09
	cca-miR1438g	GGUACUUUUAUCAUUUUUAA		20	168	76.79	0.92	0.35	0.25	0.09	4.76
1438	cca-miR1438h	UUAAAAAUAAUAAUAUUACCCU	AAU	22	393	76.59	0.62	0.32	0.40	0.13	5.85
1439	cca-miR1439a	UUUUGGAACGGAAUGAGUA	AAU	19	111	76.58	0.65	0.31	0.12	0.04	7.21
1446	cca-miR1446a	UUGAGAGAGAGAAUUCAGAA	UCU	20	505	63.17	1.01	0.34	0.20	0.06	4.55
1507	cca-miR1507a	UCAUUCCAAACGUCAUCU	CAU	18	242	65.70	0.55	0.29	0.30	0.10	4.13
	cca-miR1508a	UAGAAAGUGAAAUAGCAGUUG		21	392	56.63	0.53	0.29	0.30	0.10	3.06
1508	cca-miR1508b	UAGAGAGGGAAAUGGCAGUUG	UUG	21	88	63.64	0.89	0.38	0.12	0.05	6.82
1510	cca-miR1510a	UGUUGUUUUACCUAUUCCACC	UGU	21	92	61.96	1.19	0.34	0.13	0.04	5.43
	cca-miR1511a	CAUGGUAUCAGAGCUUGGUU		20	344	63.66	0.59	0.28	0.39	0.11	2.91
1511	cca-miR1511b	AACCAAGCUCUGAUACCAUG	CAU	20	241	67.63	0.43	0.27	0.44	0.14	3.32
	cca-miR1512a	AUACAUUAAGAAUUUUUAGUU		21	356	76.97	1.38	0.37	0.13	0.05	5.62
1512	cca-miR1512b	UAACUGAACAUUCUUAAAGCAU	AAU	22	488	69.06	0.58	0.29	0.43	0.13	3.28
	cca-miR1514a	CAAUGCCUAUUUUAAAAAUGAA		22	66	66.67	1.04	0.29	0.16	0.05	3.03
1514	cca-miR1514b	UUCAUUUUAAAAUAGGCAUUG	UUU	21	79	67.09	1.08	0.33	0.16	0.06	3.80
	cca-miR1516a	CAAGUUAGAAGCACUUUUGAGAG		23	94	68.09	0.94	0.38	0.09	0.04	4.26
	cca-miR1516b	UAUUUUCAGUAGAGAAGCU		19	97	70.10	0.86	0.37	0.16	0.06	3.09
	cca-miR1516c	AGCUUCUCUACUGAAAAUAUA		21	111	69.37	1.26	0.39	0.03	0.02	2.70
1516	cca-miR1516d	UUGGAUACAAGCUAGAAGUAC	CUU	21	129	67.44	1.06	0.36	0.21	0.08	3.10
1518	cca-miR1518a	UGUGUAGUAAAUUGAAUAUCA	ACA	21	275	74.18	0.69	0.30	0.34	0.11	2.91
1520	cca-miR1520a	CAAUGAGAACAUGUCACAUGACAA	UGA	24	380	61.84	0.81	0.34	0.26	0.09	2.63
1521	cca-miR1521a	UCAACAUUUUUCAUUAAC	AUU	18	406	61.08	0.41	0.25	0.42	0.12	3.20

	cca-miR1521b	UGUUAUUGGAAAAUGUUG		18	113	76.11	0.80	0.32	0.21	0.07	3.54
	cca-miR1521c	AACAUUUUCCAGUAACAG		18	424	71.23	0.80	0.32	0.41	0.12	4.01
	cca-miR1522a	AUUUCAUCUUAUGCAAUAA		19	385	76.88	0.65	0.29	0.41	0.12	4.42
	cca-miR1522b	AUUUCAUUUUAAGUAAUAA		19	177	75.14	0.80	0.31	0.32	0.11	3.95
	cca-miR1522c	AUUUUAUUUUAAACAAUAA		19	156	76.92	0.60	0.28	0.46	0.14	7.69
	cca-miR1522d	UUUAUUUCUUAAAAUUAAAU		20	497	64.59	0.76	0.27	0.25	0.07	3.22
1522	cca-miR1522e	UUUAUUGCUGAAAAUGAAA	UAA	19	477	75.68	0.66	0.31	0.33	0.10	2.94
	cca-miR1525a	UGGGUUAAAUAAGUUUUUAGU		21	443	73.14	0.57	0.28	0.48	0.13	4.97
1525	cca-miR1525b	ACUAAAAACUUAUUUAACC	UAA	19	144	75.00	0.66	0.33	0.38	0.13	6.94
	cca-miR1527a	GGUUUUGUAGGGUUGAGUUA		20	145	73.10	0.46	0.29	0.40	0.12	2.76
1527	cca-miR1527b	UAACUCAACCCUACAAAACC	AAC	20	76	60.53	0.94	0.32	0.15	0.06	3.95
	cca-miR1530a	UUUUCACAUAUAUUAAUAUAU		21	475	72.84	0.74	0.33	0.35	0.11	5.47
	cca-miR1530b	UAUUUUCAUUUAUGUUAAAA		20	232	74.14	0.53	0.29	0.41	0.12	3.02
	cca-miR1530c	AUAUUAUAAUUUCUGUGAAAA		21	410	73.66	0.74	0.29	0.32	0.10	4.15
	cca-miR1530d	AUAUUGUACUUUAUGUGAAA		20	186	65.05	0.65	0.33	0.36	0.12	4.30
	cca-miR1530e	UUUUAACAUAAAUUAAAAUAU		21	504	76.98	0.69	0.30	0.36	0.10	5.16
1530	cca-miR1530f	AUAUUUUUAUUUAUAUGAAAA	UAU	21	111	75.68	0.56	0.32	0.40	0.12	6.31
	cca-miR1533a	UCAUUAUUAUUUUAAUUAU		19	498	70.08	0.64	0.29	0.23	0.07	4.22
1533	cca-miR1533b	AUAAUAAAAAUAAUCAUGA	UAU	19	205	74.15	0.82	0.33	0.15	0.05	4.88
	cca-miR1534a	UAUUUUGAGUAAAUGGUCA		19	503	70.58	0.70	0.29	0.43	0.13	4.17
1534	cca-miR1534b	UAUUUUCGGUGAAUAGUCAU	AUU	20	418	76.56	0.78	0.32	0.35	0.10	6.94
1535	cca-miR1535a	UUGUUUGUGGUUAUGUC	CUU	17	108	61.11	0.73	0.31	0.11	0.03	3.70
1852	cca-miR1852a	UAUGGAUUCUGAUUGCAGGU	AUU	20	142	61.27	0.67	0.31	0.21	0.08	2.82
1854	cca-miR1854a	UGGUGAAAUUUGGAGCUUGGA	AUU	21	430	60.70	0.60	0.30	0.48	0.14	3.49
	cca-miR1863a	AAAUCUAACAUAGUAUCAGAGC		22	423	65.72	0.72	0.31	0.39	0.13	2.60
	cca-miR1863b	AGCUCUGAUACCAUCUUAAAUUU		23	126	65.87	0.80	0.32	0.25	0.08	3.17
1863	cca-miR1863c	GCUCUGAUUCCAUGUUAGAUU	UGA	21	486	68.52	0.64	0.31	0.28	0.09	3.09
1916	cca-miR1916a	UUCACUUUGACACCUCAA	UGA	18	498	72.49	0.92	0.35	0.21	0.07	3.61
1917	cca-miR1917a	ACUUUAGUAUUCUUUAUUAAU	UAA	21	253	75.49	0.64	0.30	0.44	0.13	4.35
2055	cca-miR2055a	UCCUUGGGAAGUUGGUUUC	AAG	19	258	62.40	0.45	0.29	0.41	0.13	2.71
2079	cca-miR2079a	AGAGUUGAUGUUGAUGAC	UAA	18	238	66.81	0.65	0.30	0.43	0.13	3.78

2086	cca-miR2086a	GACAUGAAUGCAGAACUG	UUU	18	116	67.24	0.77	0.31	0.24	0.08	5.17
	cca-miR2089a	AUGGUAUUGGUGGAAUUGGUAA		22	369	61.79	0.67	0.33	0.28	0.10	2.71
2089	cca-miR2089b	AUGGAAUCGGGGGAAUAGGUAA	AUU	22	466	67.38	0.67	0.32	0.46	0.15	4.29
	cca-miR2090a	AAAAAUUCUAAAAUCAGAGU	AUU	20	505	73.47	0.85	0.34	0.31	0.10	5.94
2090	cca-miR2093a	UGCAUGAAUUGGAAGAUG		18	496	73.19	0.66	0.27	0.37	0.11	4.84
2093	cca-miR2093b	UGUUCUUUCUAUUAAUGCA	AUU	19	480	60.42	0.71	0.33	0.41	0.13	2.71
2101	cca-miR2101a	ACAUUUUUACAAGAUAAAAUGU	CAA	22	270	70.00	0.50	0.29	0.32	0.10	3.70
2102	cca-miR2102a	GUGGCGGCGGAUGCUUGCCC	CCG	20	110	39.09	0.75	0.37	0.11	0.04	2.73
2105	cca-miR2105a	UGUGAAGUGAAUGAUUCAU	AUU	19	416	62.74	0.60	0.30	0.29	0.09	3.61
2108	cca-miR2108a	UUAAUGUGUAUGUGUUUGUGAG	UGU	22	240	60.00	0.76	0.32	0.25	0.08	5.00
	cca-miR2111a	CAAACCUUAGGAUGAAGAUU		20	112	66.96	0.69	0.33	0.07	0.03	3.57
	cca-miR2111b	GUCCUUGGGAUGCAGAUUACC		21	227	62.11	1.05	0.35	0.21	0.07	2.64
2111	cca-miR2111c	UAAUCUGCAUCCUGAGGUUUA	UUA	21	87	56.32	1.19	0.39	0.08	0.04	3.45
2118	cca-miR2118a	UAGGAAUGGGUGGAAUCGG	UCC	19	160	56.88	0.57	0.26	0.33	0.11	2.50
2119	cca-miR2119a	CAAAGGGAGUUGUAGGGGAA	UUU	20	259	67.18	0.82	0.35	0.16	0.05	4.63
2199	cca-miR2199b	UGAUACACUAGUAUGGAUCAC	AUA	21	505	72.67	0.69	0.31	0.38	0.12	3.37
2275	cca-miR2275a	AGGAUUAGCGGGACUUGAA	UUG	19	213	60.09	0.57	0.33	0.23	0.08	2.82
	cca-miR2592a	AACAACAAGACUCAAACAUUU		21	350	60.86	0.66	0.33	0.31	0.10	2.57
	cca-miR2592b	AGUAAUGCAAACUUGUUAA		19	417	65.23	0.58	0.27	0.42	0.12	2.88
	cca-miR2592c	UUUAACAAUUUUGAAUUACU		20	344	72.09	0.62	0.31	0.47	0.14	4.36
2592	cca-miR2592d	AAUGCUUAAGGCAUGUUGUU	AUU	20	360	63.89	0.64	0.34	0.37	0.12	2.50
	cca-miR2595a	AGACAAAAAGAAGAAAAUG		19	294	73.47	0.78	0.33	0.31	0.10	3.74
2595	cca-miR2595b	ACAUAAAGAAAAAAAUGUA	UUA	19	291	75.95	0.79	0.33	0.24	0.08	3.44
2600	cca-miR2600a	GCAUUGUGAGUGGCUAAUGU	UUG	20	188	68.09	0.74	0.35	0.26	0.08	5.32
2604	cca-miR2604a	UAAUUUUUGUGUGGAAGUGUU	UUA	21	116	69.83	0.73	0.34	0.12	0.05	3.45
	cca-miR2606a	AAAAGCACUUAAGGAAUUG		19	437	66.13	0.84	0.35	0.40	0.13	4.58
2606	cca-miR2606b	AAAAUCACCUAGGGAAUUGUA	AAU	21	406	67.73	0.89	0.33	0.12	0.04	3.69
	cca-miR2607a	AUGUGAUUAUGAGACAAGUG		20	352	62.50	0.74	0.34	0.34	0.11	5.40
2607	cca-miR2607b	AUGUUAUUAUGUUAUAAGUG	AUG	20	435	70.80	0.69	0.31	0.42	0.13	2.99
2608	cca-miR2608a	UUGUUCAUAUAUCAUUACUCU	UCU	21	184	67.39	0.54	0.27	0.27	0.09	2.72
2611	cca-miR2611a	UAUUUGUCAGUAUUUGUUGAA	UUG	21	458	65.07	0.83	0.34	0.29	0.09	3.93

	cca-miR2628a	AUGAAAGAAAGAUGAGUA		18	488	62.09	0.46	0.25	0.44	0.12	2.66
2628	cca-miR2628b	UUACUCAUUAUUCUUUCA	AUU	18	348	68.97	0.61	0.28	0.43	0.13	4.31
	cca-miR2630a	AAAUAACAAGGACCAAAACCA		21	488	71.72	0.69	0.31	0.41	0.13	3.07
2630	cca-miR2630b	UGGUUUUGAUCCUUGAUAUUU	AAU	21	490	66.12	0.67	0.34	0.40	0.13	3.27
	cca-miR2641a	AUAAAAGUAAAGGAUCAAA		19	487	76.80	0.98	0.35	0.30	0.10	5.54
2641	cca-miR2641b	UUUGAUCCUUUACAUUUAU	AAA	19	234	65.81	0.51	0.32	0.45	0.14	2.56
2642	cca-miR2642a	AUGAAUUUCAUCAAAACAUG	UUU	20	190	76.84	0.70	0.33	0.35	0.11	3.16
2646	cca-miR2646a	CAUGACAUAUUAGUGAUGAUGU	AUU	22	221	76.47	0.58	0.29	0.50	0.14	7.69
	cca-miR2650a	AACUUAAAUAUGAUUUUAGUCC		22	158	76.58	0.96	0.35	0.48	0.14	8.23
2650	cca-miR2650b	GGACUAAAAAUAUAUUUAAGUU	UUU	22	200	76.50	0.51	0.25	0.33	0.10	5.00
	cca-miR2655a	UAAAGUUAAGGGACCAAAAC		20	337	76.85	0.86	0.34	0.40	0.12	4.75
2655	cca-miR2655b	UUUUGGUCCCUUAAAUUUA	UAA	19	313	77.00	1.09	0.33	0.23	0.08	6.39
2657	cca-miR2657a	UGUUAUUUCAUCUUUUUUGUU	AUU	21	490	71.84	0.51	0.27	0.44	0.13	4.29
	cca-miR2665a	UGAUUUCCGGUCAAGAAUU		19	75	54.67	0.53	0.28	0.24	0.08	4.00
2665	cca-miR2665b	AUUUCAGGGCAAGAAUUGA	GAA	19	111	59.46	0.45	0.29	0.34	0.11	2.70
2671	cca-miR2671a	UUAAAAGUUUAGUUUUGGUC	UAA	20	271	75.28	0.71	0.31	0.30	0.11	5.17
	cca-miR2673a	GUGGAAGAGGAAGAGGAAGAGG		22	107	57.94	0.65	0.38	0.49	0.16	2.80
2673	cca-miR2673b	UCUUCCUCUUCCUCUACCAC	UUG	20	400	56.25	0.57	0.29	0.33	0.10	3.75
	cca-miR2676a	AAAUUAUUAUCCCAACAAU		19	333	71.17	0.46	0.29	0.44	0.14	3.60
	cca-miR2676b	CAUUGUUUGGAUAUUAAUU		19	142	76.06	0.67	0.35	0.25	0.09	4.93
2676	cca-miR2676c	AUUGUUUUGAAAAUAAUUUG	AAU	20	76	73.68	0.82	0.36	0.08	0.03	3.95
2866	cca-miR2866a	GAUGCUUAACAAAAACUAGA	AAC	20	96	76.04	0.95	0.38	0.16	0.07	3.13
	cca-miR2868a	UUUCUUCUACACAAAACCA		19	463	71.06	0.82	0.37	0.34	0.10	3.46
2868	cca-miR2868b	UUGGUUUUGUGUAAUAGAA	UUG	19	83	75.90	0.48	0.34	0.40	0.12	3.61
2871	cca-miR2871a	UAUUUUAGUCUUUAUGGUCA	UAG	20	313	77.00	0.83	0.30	0.30	0.09	2.56
	cca-miR2873a	UUGGACAUGAGAUUUGGUA		19	348	65.23	0.47	0.27	0.35	0.11	2.59
	cca-miR2873b	CAAAUGAAGUUGAGUUUG		18	499	59.12	0.63	0.30	0.42	0.13	3.01
2873	cca-miR2873c	CCAAACUCAGCUUCAUUUG	UUU	19	467	62.10	0.64	0.30	0.36	0.12	2.57
2878	cca-miR2878a	AGGAUUUUAUAUGUAAAGAAU	AUA	21	369	70.46	0.66	0.32	0.51	0.14	4.34
2905	cca-miR2905a	ACAUGUCAAUGACAAAGGC	UGU	19	397	71.28	0.54	0.30	0.33	0.10	3.02
2912	cca-miR2912a	UCUAGAACUCCAGAUAUGG	UCU	19	335	55.52	0.73	0.37	0.30	0.10	2.69

2920	cca-miR2920a	AAACAACGAUAUAAUAUUUCAAA	CAA	23	459	71.90	0.63	0.34	0.48	0.15	2.61
2923	cca-miR2923a	UUUGGUAUUUAAAUUUUUGUCU	AUA	22	234	73.93	0.72	0.29	0.28	0.09	3.85
2928	cca-miR2928a	AAGAAGAGGACAUUUUGU	UUU	18	277	61.01	0.61	0.31	0.42	0.13	2.53
	cca-miR2931a	CUUUAUUGUGGAUGUCAA		18	432	70.60	0.65	0.29	0.36	0.10	2.78
	cca-miR2931b	UUAUUGUUGAUGUUAAAA		18	303	72.28	0.82	0.35	0.40	0.12	3.63
2931	cca-miR2931c	UUUUGACGUCAAAAAUAAA	UAU	19	497	73.24	0.60	0.27	0.52	0.15	3.62
3433	cca-miR3433a	GUCAUGUGAGUACUGUGAUG	AUG	20	504	58.33	0.60	0.32	0.43	0.13	3.37
	cca-miR3434a	GGUUGAUUCUCUGAUUUUG		19	502	75.10	0.75	0.30	0.30	0.09	4.18
3434	cca-miR3434b	UCAAAAUAAGAGAAUAAACC	UAA	20	505	76.44	0.73	0.29	0.46	0.14	5.35
3436	cca-miR3436a	AUCAACAAUGAUUCUUGCUC	UUU	20	100	70.00	0.41	0.25	0.27	0.08	3.00
3438	cca-miR3438a	UCAAGGAUUUGGCUAUGAAG	UUU	20	338	70.71	0.69	0.26	0.42	0.11	3.25
3441	cca-miR3441a	GAAGAAGAUGAAGUCGUUUU	GAA	20	489	67.69	0.64	0.31	0.35	0.11	2.86
3444	cca-miR3444a	AUCUCAUCAAUGUCCUAAUA	AUC	20	140	72.14	0.56	0.27	0.37	0.11	4.29
3447	cca-miR3447a	UUUGAGUGUGUUUGAUAUG	UGA	19	69	65.22	0.67	0.36	0.16	0.07	4.35
3512	cca-miR3512a	UCUAUUUGUCAUCACUUGC	UUA	19	365	69.32	0.80	0.34	0.28	0.09	3.29
	cca-miR3513a	UACAAAUUCUAUAUUAUCAA		20	436	75.92	0.88	0.32	0.41	0.13	3.21
	cca-miR3513b	UACAAUUUUUAUCUUCUCAA		20	119	70.59	0.79	0.31	0.11	0.04	3.36
	cca-miR3513c	UUGAUAAUAUAGAUAUUGUAU		21	477	73.58	0.79	0.33	0.32	0.10	3.35
3513	cca-miR3513d	UUAAAUUCUGAGUUCGUCAU	UUG	20	503	64.41	0.61	0.31	0.33	0.10	2.58
3515	cca-miR3515a	AAUGUAAAAAAUGAUCGGUAU	AAA	21	383	70.24	0.65	0.32	0.48	0.15	2.87
3522	cca-miR3522a	UGAGACCAAAUCAGCAGCUG	GCU	20	374	58.29	0.68	0.30	0.43	0.14	4.81
3626	cca-miR3626a	GUGGUUGCUUUGAAAUUGAAG	AGU	21	321	69.47	0.67	0.32	0.23	0.07	3.43
3627	cca-miR3627a	UUGUCGCAGGAUAGAGGGCACU	UGU	22	135	60.74	1.23	0.39	0.16	0.06	5.19
3629	cca-miR3629a	UUGGUUGCUGAGAAAAUGC	UUU	19	372	62.37	0.68	0.31	0.35	0.11	4.03
3630	cca-miR3630a	UUUGGGAAUCUCUCUGAUGCA	UGA	21	474	70.04	0.60	0.28	0.43	0.12	2.53
3631	cca-miR3631a	UAUAUUGUAUCAUGUCAACAA	UAA	21	135	71.85	0.83	0.37	0.17	0.06	5.19
3704	cca-miR3704a	GGUCUAGGAGGACUUGGAAAAA	AGA	22	414	62.32	0.65	0.28	0.41	0.13	2.66
	cca-miR3711a	UGGCGCUAGAAGGAGGGCCU		20	57	50.88	0.56	0.32	0.14	0.06	3.51
3711	cca-miR3711b	AGGCUCUCUUUCUAGCGCCA	ACC	20	156	57.05	0.45	0.30	0.41	0.13	2.56
3712	cca-miR3712a	GGGAGUCUGAACUUGAUCAC	AUC	20	431	73.78	0.66	0.30	0.55	0.16	2.55
3950	cca-miR3950a	UUUUUAGGCAACAUAAUUUC	UUU	20	138	76.81	0.42	0.29	0.32	0.10	5.80

	cca-miR3951b	UUUCUCUCAUAUUUAUCUA		19	101	55.45	0.68	0.34	0.27	0.09	3.96
3951	cca-miR3951c	AGAUAAAGAUGAGAAAAAAA	UCU	20	504	64.88	0.86	0.32	0.37	0.11	3.17
3979	cca-miR3979a	UUCAAGGGAGAGAGAGA	AAG	17	437	62.01	0.56	0.31	0.59	0.17	3.43
	cca-miR4223a	UGUUUAUUAAUCAAAAUCCAU	AAA	21	465	74.41	0.76	0.34	0.28	0.09	3.23
4223	cca-miR4233a	AGUUGAAGUUGAUGAUGUG		19	344	71.51	0.70	0.32	0.45	0.14	4.07
4233	cca-miR4233b	GGAGUUGUUGUUGAUGGUGUG	UGU	21	313	56.87	0.61	0.33	0.34	0.11	4.47
4237	cca-miR4237a	GAUUAUAUAUGUUAACGUUU	AUA	20	284	76.76	0.65	0.32	0.42	0.14	4.58
4238	cca-miR4238a	UUGGGAUUUCAAUUUGCAAAAA	UUU	22	312	75.00	0.70	0.30	0.50	0.15	6.73
4244	cca-miR4244a	UUGUUGAUUCCUUGUAGAU	UUG	19	214	65.89	0.55	0.31	0.37	0.12	3.74
	cca-miR4245a	AUUGUCAGUGUAAAACUUU		19	391	76.98	1.18	0.36	0.22	0.07	5.12
	cca-miR4245b	ACAAAGUUUUAUACUAACAA		20	289	76.47	0.96	0.34	0.17	0.06	4.84
4245	cca-miR4245c	AUUGACAGUAUAAAAAUUUGU	AUU	21	218	75.69	0.75	0.33	0.41	0.13	5.05
	cca-miR4246a	UUAAUCAAUAAAAAUUGGAUU		21	291	73.88	0.75	0.33	0.33	0.10	3.78
4246	cca-miR4246b	AAGCAAUGAAAAUUUGAUUU	UUU	20	427	76.11	0.85	0.32	0.41	0.13	3.04
	cca-miR4248a	UGAUUGACAAAAAUAAAAUG		20	334	69.16	0.60	0.29	0.40	0.12	2.69
4248	cca-miR4248b	ACAUGUUAUUUUUGGUAAUCA	AAA	21	334	64.07	0.59	0.32	0.45	0.14	3.29
4249	cca-miR4249a	UGAAUUUGAGAAUUUUAGGUA	UUU	21	466	75.32	0.71	0.29	0.50	0.14	4.72
4340	cca-miR4340a	UGCAGAGAUAGCGACGGGCUUA	CUG	22	59	45.76	0.59	0.32	0.15	0.05	5.08
	cca-miR4371a	UCCGUCAACCACGUCAUCACUU		22	242	66.94	1.11	0.37	0.17	0.06	3.31
4371	cca-miR4371b	AAGUGAUGACGUGAUAGACGGA	UGA	22	419	72.08	1.32	0.38	0.20	0.08	3.34
	cca-miR4376a	ACGCAGGAGAGAUGACGCUGU		21	112	50.89	1.03	0.38	0.03	0.01	4.46
4376	cca-miR4376b	CAGCGUCAUCUCUCCUGCGU	GCA	20	131	51.91	1.02	0.37	0.03	0.01	5.34
	cca-miR4413a	CAGUGACUGACAAUUCUC		18	117	63.25	0.55	0.27	0.27	0.09	5.98
4413	cca-miR4413b	AAGAGAAUUGUAAGUCACU	AUU	19	144	64.58	0.65	0.30	0.15	0.06	4.17
4414	cca-miR4414a	GAACCAACGAGUCAGCAGCU	GCU	20	117	53.85	0.59	0.28	0.23	0.07	2.56
	cca-miR4415a	CAUUGAUUCUCAUCACAAC		19	100	68.00	1.32	0.34	0.04	0.02	6.00
4415	cca-miR4415b	AAGUUGUGAUGGGAAUCAAUG	AUC	21	123	64.23	1.30	0.36	0.03	0.01	4.88
	cca-miR5031a	AAUGAUAAACAUCUAAUUU		19	345	67.25	0.64	0.30	0.31	0.10	2.90
	cca-miR5031b	AAUUAGAUGUUAAUAAUUA		19	479	66.18	0.72	0.33	0.38	0.13	2.92
	cca-miR5031c	UUAAUGAUUAAAAUAUAAUUU		21	399	76.94	0.80	0.30	0.43	0.13	6.27
5031	cca-miR5031d	AAAUUAGAAGUUACUCAUUAA	UAA	21	70	75.71	0.88	0.29	0.24	0.09	5.71

5039	cca-miR5039a	AUGCAUCGUUUAAAAAAGGG	AUU	20	262	66.41	0.48	0.29	0.50	0.14	3.82
5040	cca-miR5040a	AUGAUAAAUCACAAGCAUGA	AUA	20	318	75.79	0.75	0.28	0.24	0.07	3.46
	cca-miR5040b	UCAUGCUUGUUUUAUAUCA		19	434	76.04	0.89	0.35	0.38	0.13	3.23
5041	cca-miR5041a	UUGAUCAAGUUUAAGAUGAA	UUG	20	319	72.41	0.80	0.31	0.44	0.13	4.70
	cca-miR5041b	UUUCUUCUCCAACUUGCUCAA		21	402	74.38	0.72	0.31	0.28	0.09	3.48
5054	cca-miR5054a	UCCCCACGGUGGGCGCCA	GAU	18	443	51.47	0.66	0.34	0.52	0.16	2.93
	cca-miR5054b	UGGCGCCCACCGUGGGGA		18	80	50.00	0.65	0.30	0.20	0.07	2.50
5055	cca-miR5055a	UCGCUCCUGAGCUCGGCGU	CUG	19	278	44.24	0.65	0.32	0.48	0.16	2.52
5057	cca-miR5057a	AAUUUUAAAUCAUUUUGAC	AAA	19	433	76.67	0.99	0.34	0.12	0.05	4.85
	cca-miR5057b	UCAAAAUGAUUUCAAAUUU		19	500	77.00	0.96	0.34	0.27	0.09	3.20
5075	cca-miR5075a	CUCCGCCGCCGCCGUCCGC	CCG	19	388	27.58	0.68	0.35	0.19	0.07	5.67
5139	cca-miR5139a	UGGUAUUAGAGCCAGGUU	UAU	18	120	70.00	0.81	0.32	0.16	0.06	5.83
5140	cca-miR5140a	GCUGGUGAAAAUUUGGUG	UGG	18	287	62.37	0.71	0.33	0.32	0.10	2.79
5142	cca-miR5142a	AUCACAUAUCAAUCAAUAU	UCA	19	248	70.16	0.59	0.29	0.33	0.11	2.82
	cca-miR5142b	AUAUUGUUUUAUAAGUGAU		19	459	72.98	0.78	0.32	0.37	0.11	3.70
5163	cca-miR5163a	CCUAUCCUAAAAUAUUUAAAA	AUU	21	279	67.38	0.49	0.29	0.44	0.13	3.23
	cca-miR5163b	UUUUAAAUAUUUUAGGCUA		19	161	75.16	0.72	0.36	0.34	0.11	5.59
	cca-miR5163c	CACCUAACUGAAAUAUUUA		19	413	69.73	0.64	0.30	0.31	0.10	3.63
5171	cca-miR5171a	UUCUUCCAUCCCAUAAUAAGU	AUU	21	453	76.82	0.82	0.30	0.38	0.12	5.74
5174	cca-miR5174b	UUAUGGAACAAAGGGAGUA	UUU	19	152	76.97	0.81	0.30	0.13	0.05	5.26
5183	cca-miR5183a	UUUGGACAAAUUUGUGUCA	AUA	19	99	70.71	0.79	0.35	0.20	0.09	4.04
5185	cca-miR5185a	GCUUCUAAUUCAAUUCUCAAA	UUC	21	148	76.35	0.69	0.34	0.28	0.09	2.70
	cca-miR5185b	UUCUAGUUUAUUUUUCAAA		19	91	76.92	0.80	0.32	0.11	0.04	4.40
	cca-miR5185c	AUUUAGAGAUAGACUUAGA		19	143	61.54	0.42	0.27	0.53	0.16	2.80
	cca-miR5185d	UUCUAAGUCCUUCUCUAAAUC		21	465	65.16	0.56	0.26	0.23	0.07	2.58
	cca-miR5185e	UUUGAUAAUUGAACUUGAAG		20	197	64.97	0.41	0.28	0.41	0.12	5.08
5201	cca-miR5201a	UUGAUCUUUUGCCUCACCCU	UGA	20	377	58.89	0.63	0.31	0.42	0.14	2.92
5205	cca-miR5205a	CUUAUAAUUAGGGAUGGAGGGAGU	GGA	24	200	73.00	0.55	0.33	0.44	0.14	2.50
5219	cca-miR5219a	UCAUGGAAUUUCUGCUGCUGCA	AGG	22	74	55.41	0.80	0.34	0.30	0.13	2.70
5234	cca-miR5234a	UUUUGUUAUGGAUGGCUGAAG	AUU	21	387	62.79	0.67	0.31	0.42	0.13	4.13
5237	cca-miR5237a	UCAAAAAAUUUAGUUGGGA	AUU	19	268	62.69	0.56	0.33	0.38	0.11	3.36

	cca-miR5240a	UUGAAAAAAUUUUGGGUUUG		20	153	72.55	1.10	0.35	0.11	0.05	2.61
5240	cca-miR5240b	AAAUCCCCAAUUUUUUCAA	AAU	19	424	76.89	0.58	0.29	0.48	0.14	5.42
5248	cca-miR5248a	UUUUUAGUUGGCAUGCAUUCA	UUG	21	153	61.44	0.69	0.36	0.27	0.10	4.58
5253	cca-miR5253a	UGAAAAUGAUUGUGUUGGA	GAU	19	212	60.38	0.56	0.27	0.39	0.12	3.30
	cca-miR5255a	UGACUUGAUAGAGGACAUGGG		21	500	65.00	0.71	0.32	0.40	0.12	2.60
5255	cca-miR5255b	CCCAUGUUCUCUAACAAGUCA	AUG	21	292	65.75	0.47	0.30	0.43	0.14	2.74
	cca-miR5256a	UUAAUCUUACAUAAUCCAUUA		21	372	74.73	0.68	0.32	0.31	0.10	3.76
5256	cca-miR5256b	UAAUGGAUUAGAUAAGAUUAA	AAU	21	168	76.19	0.67	0.35	0.47	0.15	7.74
	cca-miR5257a	AAGUAGAACCUUUUUUCUG		19	235	71.06	0.94	0.34	0.15	0.05	2.98
5257	cca-miR5257b	CAGAAAAAAGGUAUCUACUUGU	AAG	22	479	54.49	0.66	0.31	0.47	0.14	3.55
	cca-miR5260a	AAGCCAUGUGAACAAUACA		19	255	64.31	0.49	0.26	0.37	0.11	3.53
5260	cca-miR5260b	UUUGUAUUGUUGCAAUGGCUU	CAA	21	423	71.39	0.61	0.32	0.38	0.11	2.84
5261	cca-miR5261a	UCAUUGUAAAUGGCUUUGGCU	AAU	21	364	64.29	0.54	0.27	0.30	0.09	3.85
5264	cca-miR5264a	UUGAUCAUGGACUUUGCA	UUG	18	111	61.26	0.69	0.32	0.26	0.10	5.41
5265	cca-miR5265a	AAGUGAUGUUGGAAUGGU	GUU	18	212	63.68	0.57	0.27	0.43	0.12	2.83
	cca-miR5281a	CUCCCUCCGGUCCUUUUAAUAAGA		24	94	64.89	1.54	0.39	0.03	0.01	3.19
5281	cca-miR5281b	UCUUAUAUAAAGGACUGGAGGGAG	AAU	24	395	73.92	0.97	0.32	0.34	0.10	4.81
5285	cca-miR5285a	UGGGACUUAGGGUAGGAUUAGGCG	UUG	24	136	72.06	0.62	0.29	0.40	0.14	4.41
5288	cca-miR5288a	CAGCUUUGAAGAACAUAGGAAUUA	GAA	24	362	61.60	0.61	0.28	0.49	0.14	3.31
5291	cca-miR5291a	GUUUGAGGGAAGGAUUGGUUGGAU	GAU	24	437	75.29	0.71	0.29	0.21	0.06	2.52
5292	cca-miR5292a	UCUUUGUUGCUCUCAGCUGAAUC	GAU	23	230	59.57	0.64	0.31	0.40	0.12	2.61
5368	cca-miR5368a	GGACAGUCUCAGGUAGACA	AGA	19	172	45.35	0.77	0.34	0.33	0.11	2.91
	cca-miR5369a	UGAGAAAAGGAGAAUGU		17	118	71.19	0.79	0.36	0.15	0.05	3.39
5369	cca-miR5369b	UGAGAUAAUGAGGAUGUCA	UGA	19	59	71.19	0.66	0.34	0.19	0.06	8.47
5372	cca-miR5372a	UGUUUGAUAAAACUGUUGU	UGU	19	454	75.55	0.70	0.34	0.57	0.16	4.63
5373	cca-miR5373a	UCUCUUGAUUCUAGAUGAUGU	AGA	21	466	64.59	0.62	0.30	0.44	0.13	3.00
5374	cca-miR5374a	UUAUAAUCCGACAUCUGGAAU	AAU	21	330	63.94	0.56	0.29	0.49	0.14	4.55
5379	cca-miR5379a	GAUAUCAAAAGGGAUGAUUUUCAU	AUU	24	341	76.54	0.71	0.33	0.45	0.14	4.11
5382	cca-miR5382a	AGGGCCUGUUUAGAU	AAA	15	260	74.23	0.89	0.35	0.20	0.07	4.23
	cca-miR5512a	GAUAUGGUAAUGCUAAAA		18	444	72.30	0.57	0.29	0.35	0.10	6.76
5512	cca-miR5512b	UUUUAGCAUUACCAAAUCCUA	AAU	21	71	73.24	0.85	0.37	0.21	0.08	5.63

5521	cca-miR5521a	AUCAGAUAUGCAGCCACU	UGA	18	465	66.24	0.81	0.31	0.31	0.10	3.44
5523	cca-miR5523a	UGAGGAGGAACGUAUUUACUAG	AUU	22	80	71.25	0.91	0.30	0.06	0.02	2.50
5532	cca-miR5532a	AUGGAAUAUAUGACAAAGGU	UAU	20	234	73.93	0.72	0.33	0.49	0.14	7.26
5555	cca-miR5555a	CAAAGUAAUAUUAUUCUCUU	UAU	20	429	73.19	0.61	0.29	0.35	0.12	5.36
5558	cca-miR5558a	GAUAGACUUAUAAUUUGAAA	AUU	20	384	68.49	0.52	0.28	0.38	0.11	4.17
	cca-miR5559a	UACUUAGUGAACUGUUGGAUC		21	346	63.87	0.49	0.30	0.29	0.09	3.76
5559	cca-miR5559b	GAUCCAACAAUUCACUAAGUA	AUU	21	181	66.30	1.02	0.34	0.13	0.05	2.76
5561	cca-miR5561a	UCCAUUUAGUGAGAGAAAGAC	AAU	21	283	74.56	0.72	0.34	0.34	0.12	3.53
	cca-miR5565a	UUGUUUGAAUGAUGUCGGA		19	252	76.98	0.56	0.31	0.45	0.14	5.16
5565	cca-miR5565b	UUGAUAAAAAUCCGACUA	AAU	18	214	68.22	0.55	0.29	0.31	0.10	4.21
	cca-miR5568a	CAAAAGCUAUGUACCGAGAAA		21	157	76.43	0.66	0.31	0.31	0.12	8.28
	cca-miR5568b	CUUACAGUUAGGAAUGGAGG		20	504	76.79	0.77	0.32	0.34	0.11	4.96
	cca-miR5568c	AAAGUUAUGUAUGUAGAAAA		20	488	72.75	0.73	0.30	0.38	0.12	3.07
	cca-miR5568d	UCUAGAAAAGCUAAGACGU		19	237	67.51	0.62	0.30	0.49	0.16	3.80
	cca-miR5568e	GAUGUUUUGAGUUUUAUAGAU		21	487	72.07	0.69	0.33	0.24	0.08	2.87
5568	cca-miR5568f	CAAAUUUUAAAAUGUUUUGG	UAU	20	370	71.89	0.65	0.29	0.43	0.13	3.24
5668	cca-miR5668g	CAGUCUCUAAUUCCGUUGCU	AAG	20	146	72.60	0.70	0.34	0.20	0.07	2.74
5672	cca-miR5672a	CAUGGUAAUGGAAGAAAUGGA	AAU	21	146	71.23	1.10	0.37	0.32	0.10	3.42
	cca-miR5712a	CUCACCAAUUGUAUUAUUAUU		21	412	70.15	0.44	0.27	0.37	0.11	5.10
5712	cca-miR5712b	AAUAUUAAUUUAAUUAGUGA	AUU	20	465	72.26	0.77	0.31	0.27	0.09	6.24
5716	cca-miR5716a	UUUAGAUCUUCAAUUAGCCAA	UUC	21	422	69.19	0.51	0.27	0.37	0.11	2.61
	cca-miR5721a	CCAUUUUUCUCUCCAUUUUU		20	459	59.91	0.62	0.27	0.28	0.08	3.27
5721	cca-miR5721b	AAAAAUGGAGUGAGAAAU	UUU	18	60	76.67	0.92	0.32	0.14	0.04	5.00
5722	cca-miR5722a	AAAUAGAGUAAUGUGGAACG	AUU	20	471	69.85	0.71	0.34	0.30	0.10	5.52
	cca-miR5741a	AAACCAUCAAAUUGGUCCCUA		21	180	73.89	0.68	0.29	0.38	0.10	5.00
5741	cca-miR5741b	UAGGGACUAAAUUGAUGGUUU	AUU	21	278	69.42	1.23	0.36	0.15	0.05	4.68
5745	cca-miR5745a	UUUAAUUUAUAUGCAUCGUCA	CAU	21	78	76.92	0.72	0.32	0.20	0.07	3.85
5757	cca-miR5757a	UAGAUAUUUGUUUAACAGC	AAU	19	398	75.63	0.73	0.29	0.45	0.13	5.28
5770	cca-miR5770a	GGACUAUGGUUUGGACAA	UUG	18	447	52.80	0.69	0.34	0.36	0.13	2.91
5773	cca-miR5773a	ACCUAACUUAACCUUAUAAAA	AAA	21	68	67.65	0.95	0.32	0.08	0.03	2.94
5775	cca-miR5775a	AAGCGCUUUUGAGAGCUUC	UCU	19	363	72.73	1.28	0.37	0.07	0.03	2.75

5778	cca-miR5778a	CGACGAUCUCUUCGUCGACAUC	UCG	22	58	48.28	0.66	0.33	0.18	0.06	6.90
5828	cca-miR5828a	GAUUUACAUUUAUUGCCGUA	UAA	20	188	65.43	0.49	0.30	0.19	0.06	3.19
5837	cca-miR5837a	UGUUGGAAAGACAUGCAGC	AUG	19	189	68.25	0.67	0.30	0.41	0.13	4.23
	cca-miR6025a	GAUGUUGUUUCAAUUGUUGGUA		22	489	72.80	0.79	0.30	0.25	0.08	3.89
	cca-miR6025b	UCAAUUGGGAUGACUUCUAGU		21	427	62.76	0.73	0.34	0.34	0.11	4.68
6025	cca-miR6025c	AUGUCACCUCUAAAAUUGGCA	UUG	21	446	65.47	0.66	0.31	0.34	0.10	4.26
6034	cca-miR6034a	UGAUGUGUAUAGCUUUGGG	UAU	19	75	54.67	0.76	0.32	0.06	0.02	4.00
6111	cca-miR6111a	GUCAUAAAUCGUGACCUAAAG	AUG	21	491	71.69	0.68	0.32	0.32	0.10	2.65
	cca-miR6135a	GGUAAGUUGGUUAAUUGA		18	501	66.67	0.64	0.34	0.37	0.10	4.19
6135	cca-miR6135b	GAGUAAGUUGGUCAUUUGG	AUU	19	496	73.39	0.74	0.33	0.34	0.10	4.44
6140	cca-miR6140a	AUGUUUGUAAAUAGUUUGUGU	UUG	21	59	76.27	0.44	0.36	0.37	0.13	5.08
6148	cca-miR6148a	AGAACAAUCGAUCGUCGUA	GUU	19	431	64.04	0.65	0.31	0.42	0.12	2.55
	cca-miR6169a	AGAGAGAAAAUAAAAAUACU		20	147	76.87	0.82	0.33	0.41	0.13	3.40
6169	cca-miR6169b	UAGUAUUUAUCUUUUCUCU	AAU	19	468	65.38	0.80	0.33	0.20	0.06	2.56
6173	cca-miR6173a	AGUAUCCAUCGUUUACGGCU	AGC	20	113	46.02	0.53	0.33	0.25	0.08	2.65
6182	cca-miR6182a	UGAGUGUGUGAAUGAUGGCUUU	CAA	22	88	55.68	0.58	0.34	0.24	0.09	3.41
6191	cca-miR6191a	UUCAUAUCUAGAUAAAUCU	AUU	19	97	72.16	0.85	0.34	0.07	0.03	3.09
6196	cca-miR6196a	UCCUCUCCCUCUCCUCCUCCU	GAG	21	503	46.92	0.63	0.28	0.27	0.09	2.58
	cca-miR6202a	UUCAAUGUAUAAAAUCUUC		19	384	67.97	0.55	0.25	0.37	0.11	4.43
6202	cca-miR6202b	AAGAUUUUAAGAAUUGAA	UGU	18	394	72.59	0.76	0.34	0.33	0.10	2.54
	cca-miR6218a	ACAAGUUUCUUGAUUCUUGG		20	398	72.86	0.62	0.30	0.45	0.13	4.02
6218	cca-miR6218b	CCAAAAAUCAAGAAACUUG	AAA	19	471	69.85	0.62	0.28	0.35	0.11	2.97
6230	cca-miR6230a	CAAGCUUAGGGACCCAAAA	AAA	19	141	76.60	0.71	0.34	0.28	0.10	2.84
6231	cca-miR6231a	UAUUUUUGGGCUCAUGGACAU	UGG	21	156	58.33	0.63	0.31	0.19	0.07	3.85
	cca-miR6232a	CAAAUACAAUGUACCAAAA		19	235	76.60	0.65	0.27	0.42	0.14	5.11
6232	cca-miR6232b	UUUUUGGUACUUUGAAUUU	AUA	19	463	76.03	0.68	0.31	0.36	0.10	3.02
6271	cca-miR6271a	CAUUGUAUCUCUCAAUAUUGA	UCU	21	340	72.94	0.63	0.30	0.42	0.13	2.65
	cca-miR6281a	UUAGAGAGAGAGAGAGUGAG		20	454	55.73	0.67	0.32	0.32	0.10	3.52
6281	cca-miR6281b	CUCACUCUCUCUAUGUCUAA	GAG	20	378	56.61	0.55	0.26	0.18	0.06	3.17
	cca-miR6288a	AAUCAAUUAGAAACUAACAA		20	413	77.00	0.75	0.31	0.36	0.11	3.39
6288	cca-miR6288b	UUGUAAUUUUUUUAUUGAUU	UUA	20	371	73.05	0.67	0.30	0.42	0.12	2.96

	cca-miR6288c	UUGUUAUUUUAUAAUGGAUU		20	126	73.81	0.70	0.32	0.31	0.11	5.56
	cca-miR6288d	CUUGUUAAUAUUUAAUUGAU		20	130	75.38	0.83	0.35	0.15	0.06	6.15
	cca-miR6288e	CUAGCCACUUGUCCUUUUC		19	184	65.76	0.66	0.33	0.16	0.06	3.26
6291	cca-miR6291a	CUUACCAUGUUUUUAUACCA	UAU	20	81	72.84	0.81	0.27	0.20	0.08	3.70
	cca-miR6299a	AUUUAAAAUAAUUAAUUUGUCA		22	318	76.73	0.77	0.30	0.36	0.10	8.81
6299	cca-miR6299b	UGACAAUUCAAUAAUUUGAAAU	AUU	22	444	76.35	0.79	0.32	0.47	0.14	6.98
	cca-miR6300a	CCACUAUACUACAACGAC		18	452	71.68	0.78	0.33	0.31	0.10	3.10
6300	cca-miR6300b	GUCGUUGUAGUAUAGUGG	AUA	18	311	63.99	0.58	0.27	0.36	0.11	2.89
6443	cca-miR6443a	CAUCAUCUAUGAUCAUAC	AAA	18	457	62.80	0.56	0.25	0.23	0.07	4.16
6449	cca-miR6449a	CAUGGUUCUGAAUAAUGGUU	GUU	20	317	68.14	0.66	0.33	0.40	0.12	3.47
6457	cca-miR6457a	UUAGUUGGGCACCCUCUUCU	AGA	20	216	63.43	0.67	0.30	0.35	0.11	3.24
6459	cca-miR6459a	GAUCGAAUUUGGUCUUGAGA	AAG	20	363	65.84	0.74	0.34	0.52	0.16	3.31
6462	cca-miR6462a	AAGGGACAACAAUGGCCUAAG	AAG	21	123	60.98	0.74	0.31	0.16	0.05	3.25
	cca-miR6464a	UGAUUGUUUGUUGGAUAUUAU		21	277	68.23	0.56	0.34	0.38	0.11	3.61
6464	cca-miR6464b	AAUAUCCAACAAGAAAUCA	UAU	19	106	68.87	0.66	0.36	0.14	0.05	2.83
6466	cca-miR6466a	CUGGUAUGAGCAUUAGAUGA	AAU	20	428	68.93	0.63	0.27	0.36	0.11	4.67
	cca-miR6470a	UCUGAUGUCAUAUUAAAAA		19	98	71.43	0.74	0.35	0.15	0.06	3.06
	cca-miR6470b	UUUUUAAUAUGACAUCAGAG		20	408	75.49	0.87	0.32	0.48	0.15	2.70
6470	cca-miR6470c	UUUUCUAAGAUGAUAUCAGAG	UUG	21	455	66.59	0.70	0.30	0.47	0.14	2.86
6476	cca-miR6476a	AGUGGAGAUGAUACAUGA	AUG	18	476	62.82	0.63	0.29	0.31	0.09	2.94
6478	cca-miR6478a	ACCAACUGAGCUAAGGUCGG	UUG	20	180	67.22	0.58	0.31	0.41	0.13	3.33
6483	cca-miR6483a	UAUUGUAGAAAGUUUAAGGAU	AUU	21	284	73.59	0.74	0.33	0.28	0.09	5.99
6485	cca-miR6485a	UAGGAUGUAGAAGAUCAUAA	AUU	20	394	67.51	0.75	0.29	0.24	0.07	2.79
7124	cca-miR7124a	AAAUAAAGUCAAUAUUGGUG	AUU	20	464	67.24	0.64	0.32	0.47	0.14	3.45
7125	cca-miR7125a	AUUGAUCUUGUUGCACCUA	CAA	19	465	67.10	0.58	0.29	0.55	0.17	3.23
7127	cca-miR7127a	UAUGACAAAUUCUUUGAGUAU	AAU	21	182	72.53	0.68	0.31	0.38	0.13	3.85
7484	cca-miR7484a	UUUGUAUAUUAGUUCAAAUAGCA	AUU	23	226	76.11	0.62	0.32	0.40	0.12	7.08
7488	cca-miR7488a	UUUUGUUCCCUGUAGUCAAAA	AUU	21	384	67.71	0.55	0.26	0.47	0.15	3.91
7508	cca-miR7508a	CAAGAAAAGAAGUCUGGAG	CUU	19	493	51.93	0.68	0.31	0.31	0.10	3.04
7516	cca-miR7516a	GCGGGUAUCUUCGCCUCUGA	AUU	20	79	50.63	0.90	0.37	0.11	0.04	2.53
7532	cca-miR7532a	GAAGCUGCGUCUGGUGGUGGU	GGU	21	310	61.61	0.72	0.34	0.43	0.14	2.58

7534	cca-miR7534a	GCAACUUGACUAUAGUUUGAC	UUG	21	465	62.58	0.67	0.32	0.50	0.14	4.30
	cca-miR7535a	ACCACACUCACAUUUUC		17	152	63.16	0.69	0.32	0.22	0.07	3.29
7535	cca-miR7535b	GAAAAUGUGAGUGUGGU	UGU	17	497	62.17	0.61	0.29	0.46	0.14	3.42
7540	cca-miR7540a	UGAUAUGAUUAAUGAUGUGA	UGA	20	273	76.92	0.65	0.26	0.36	0.10	2.93
	cca-miR7543a	UUAAAGAUACAUAUUUGAC		19	497	76.46	0.80	0.33	0.35	0.11	3.82
7543	cca-miR7543b	UAAUUAUACAUGUUUGACU	UUU	19	389	72.49	0.62	0.29	0.55	0.15	2.57
7545	cca-miR7545a	CACUUUAGCUUUCCCAA	AUU	17	158	60.76	0.45	0.28	0.39	0.12	2.53
7696	cca-miR7696a	UCAAGUUCUCACACUUCAAA	UUC	20	208	70.19	0.48	0.27	0.43	0.13	5.77
	cca-miR7699a	GUGUAAUUAAAGCAUUAAAU		20	73	75.34	0.91	0.34	0.05	0.02	8.22
	cca-miR7699b	GUGUUAUCAAUGCAUUAAA		19	362	67.13	0.57	0.29	0.47	0.14	5.80
7699	cca-miR7699c	UUAAUGCAUUGAUUACACA	AAU	19	81	74.07	0.80	0.31	0.23	0.08	6.17
	cca-miR7701a	UUUAUAUUCAUUAAUUUAAU		20	459	74.95	0.61	0.30	0.48	0.13	4.79
	cca-miR7701b	UUUUAGAUUGAUUUAUUUAAU		21	299	75.25	0.70	0.31	0.37	0.11	4.68
	cca-miR7701c	AUUAAAUGAAUGAAUAUAAA		20	453	76.82	0.66	0.31	0.35	0.10	7.95
7701	cca-miR7701d	AUUAAGUGAGUGAAUCUAAA	AAU	20	209	76.56	0.72	0.33	0.43	0.15	4.78
7728	cca-miR7728a	CUGCUUGACUUGACUGUAUU	AUU	20	477	60.80	0.61	0.28	0.50	0.14	3.14
7736	cca-miR7736a	UGUGACAUCCUUGAUAGUAA	UGA	20	458	62.45	0.56	0.31	0.24	0.08	3.06
7741	cca-miR7741a	UCUUGAAUUUUCGCAUGCAG	CAU	20	64	57.81	0.52	0.30	0.12	0.04	3.13
	cca-miR7742a	UGUGUGAAUGAGGGAAUGA		19	289	65.05	0.71	0.33	0.32	0.10	3.72
7742	cca-miR7742a	UCAUUCCCUCAUUCACACA	AUU	19	430	52.09	0.56	0.30	0.45	0.13	4.84
7745	cca-miR7745a	AGGCUUUAGUAAUCAAGAGAU	AAG	21	361	64.82	0.61	0.30	0.48	0.15	3.05
7753	cca-miR7753a	GAUGAACAAGGCAGAAGACAU	UCU	21	446	59.64	0.54	0.29	0.54	0.16	3.81
7757	cca-miR7757a	GUAGUUGAAGUUUUUGUUUA	UAG	20	225	76.00	0.61	0.34	0.31	0.10	3.56
7767	cca-miR7767a	ACCUGCAAGCUUCUUGCUCCU	AGC	21	277	58.48	0.71	0.30	0.36	0.12	2.89
	cca-miR7776a	CAUUCAUAAUGAGUUGAAU		19	398	71.61	0.68	0.30	0.35	0.11	2.51
7776	cca-miR7776b	UUAAUUAUGAGUUGAAUGU	AUG	19	498	59.84	0.70	0.34	0.49	0.15	3.41
7812	cca-miR7812a	CACUUCAUUAAUUCAUAACAG	AAU	21	502	67.13	0.66	0.30	0.31	0.09	4.58
7814	cca-miR7814a	UAGAUAGUUUGUAUGCUUUG	AAA	20	429	69.70	0.61	0.33	0.38	0.12	2.56
7816	cca-miR7816a	AAUGUUGUUAUGAACUCUGUA	AAU	21	501	75.45	0.62	0.28	0.50	0.14	5.99
7817	cca-miR7817a	UUUGGUUGUUGUCUUGAGACA	UCU	21	84	60.71	0.74	0.33	0.09	0.04	5.95
7822	cca-miR7822a	UACCUUUUGUUCAACUUCAA	AUU	20	370	65.95	0.52	0.28	0.44	0.13	3.24

	cca-miR7822b	UUUGAAAUUGAUUAAAUGGUA		21	342	76.32	0.77	0.32	0.33	0.09	4.97
	cca-miR7823a	AUUUCAAAUACAUGCAUGCA		20	419	74.70	0.69	0.31	0.46	0.14	2.63
7823	cca-miR7823b	UGCAUGCAUGAAGUUGAAAU	AUU	20	288	72.57	0.74	0.27	0.30	0.09	3.82
	cca-miR7834a	UAAUAAAAUCUUCACUAUUAU		21	184	64.67	0.50	0.29	0.35	0.11	3.26
7834	cca-miR7834b	AUAAUAGUCAAGAUUUUAUU	AAU	20	123	67.48	0.53	0.31	0.42	0.15	3.25
7982	cca-miR7982b	AAGUUGGAUGAUAAUAAAAUAUA	AUA	23	498	76.91	1.00	0.34	0.20	0.07	3.21
	cca-miR8005a	ACUCUAAAUUCUAAAUUCUAAA		22	194	69.07	0.57	0.33	0.31	0.10	3.09
	cca-miR8005b	UUUAGGGUUUACGGUUUAGGGUUU		24	321	60.75	0.60	0.36	0.33	0.10	7.17
	cca-miR8005c	AAACACUACACCUUAAACCCUAAA		24	448	68.08	0.89	0.36	0.36	0.12	7.59
8005	cca-miR8005d	UUUAGAGUAUAAUGUUUAUAGUUU	UUA	24	260	70.38	0.67	0.36	0.47	0.14	3.85
8007	cca-miR8007a	GAAAAUUGAAAAGUGCCACAUA	UUU	22	418	74.64	0.65	0.30	0.50	0.14	4.55
	cca-miR8011a	CUCAUUUUUAUCUCAUAAAAA		21	55	76.36	1.54	0.31	0.03	0.01	3.64
	cca-miR8011b	ACUCAAUUUUGUCUCGCAAAAA		22	400	76.75	0.72	0.31	0.34	0.10	4.75
8011	cca-miR8011c	UUUUUAUGAGAGAAAAAUGAG	AAA	21	485	73.20	0.70	0.32	0.48	0.15	3.30
8014	cca-miR8014a	AUUGUUUUAUAUUUUAUUUUAUUU	UGU	24	249	76.31	0.86	0.32	0.19	0.06	2.81
8030	cca-miR8030a	AUUUGGGUUGAAUUGGUGUUAA	UUG	22	469	75.05	0.91	0.34	0.23	0.08	6.40
8035	cca-miR8035a	UCCAGCUUCAAUAUCUCUUUCU	AUU	22	407	57.99	0.63	0.30	0.33	0.10	2.70
	cca-miR8040a	GAUCAAAAUUACAAUUAUA		19	328	75.00	0.81	0.30	0.33	0.10	5.18
	cca-miR8040b	UUAUAAUUAUAUUUAUGAUC		20	112	71.43	0.84	0.33	0.10	0.04	2.68
	cca-miR8040c	UUAUAAUUGUAAUUUUGAU		19	450	76.89	0.81	0.32	0.28	0.09	8.00
8040	cca-miR8040d	GAUCAUAAUCAUAAUUAUAA	AAU	20	478	71.76	0.64	0.32	0.46	0.15	3.35
	cca-miR8041a	GCUUUGCUAAUUUCAUUG		18	231	62.77	0.59	0.31	0.41	0.12	4.33
	cca-miR8041b	GUGCAUUGCUAUUAUCAUU		19	360	61.39	0.47	0.25	0.47	0.13	3.89
8041	cca-miR8041c	AAUGAAAAUGGCAAAGCA	AUU	18	445	76.85	0.62	0.27	0.38	0.11	5.62
8044	cca-miR8044a	UUUCAAAUCUUGUUGGAGAUG	AUG	21	76	63.16	0.57	0.38	0.12	0.05	2.63
8047	cca-miR8047a	CCAUUGUUUUGAAAUUAGACC	UUU	21	264	74.62	0.84	0.31	0.27	0.09	3.41
8049	cca-miR8049a	AUCAAGCUCAUGUAGACAU	AUG	19	95	64.21	0.65	0.32	0.12	0.04	3.16
	cca-miR8051a	AAUAGUAUGGUGAAAGAAAUA		21	200	68.50	0.50	0.28	0.39	0.12	3.50
8051	cca-miR8051b	UAUUUCUUCUAACAUAUUAUU	AUU	21	199	76.88	0.61	0.31	0.44	0.13	6.03
8123	cca-miR8123a	CUUGAGCAAUGGCAC	UUG	15	215	48.37	0.71	0.34	0.36	0.12	4.19
8140	cca-miR8140a	UGAAGCUUAAGUGUUGAAAAG	AAU	21	465	76.99	0.86	0.34	0.35	0.12	5.59

	cca-miR8140b	UGAAGCUCAAGUCUUUAAAA		20	268	70.90	0.60	0.30	0.47	0.14	3.73

[a]Conserved SSR signature sequence of the miRNA family.

[b]Length of mature miRNA sequence.

[c]Length of precursor miRNA.

[d]Percentage AU content of precursor miRNA.

[e]Minimum Folding Energy Index of precursor miRNA.

[f]Normalized base-pairing propensity of precursor miRNA.

[g]Normalised Shannon entropy of precursor miRNA.

[h]Normalized base-pair distance of precursor miRNA.

[i]SSR signature repeats per 100 nucleotides of precursor miRNA.
