# Supplementary material for: Genome-wide identification of miRNAs and lncRNAs in Cajanus cajan
Source: BMC Genomics. 2017 Nov 15;18:878. doi: 10.1186/s12864-017-4232-2 (PMC5688659; doi:10.1186/s12864-017-4232-2)
Supplement: Supplementary file 2 — Predicted targets of C. cajan miRNAs. (PDF 193 kb) [file 12864_2017_4232_MOESM2_ESM.pdf]

Table S2 - Predicted targets of C. cajan miRNAs.

Family	miRNA	Target Protein	Locus (or Loci)	Mode of Action
156	cca-miR156b	Squamosa promoter-binding protein	CcLG11:33212797:33215770:1	C
158	cca-miR158b	transcription elongation factor SPT6	CcLG07:9054957:9064476:1	C
		inorganic phosphate transporter	CcLG09:4783671:4785284:1	C
		odr-4 protein	Scaffold000248:268146:273700:1	C
		serine/threonine-protein kinase	Scaffold000258:159409:161928:1	C
161	cca-miR161a	polygalacturonase	CcLG02:30091312:30103572:-1	C
162	cca-miR162d	PAZ protein	CcLG03:29072965:29085159:-1	C
164	cca-miR164e	NAC domain protein	CcLG10:7293059:7297863:-1	C
167	cca-miR167g	neutral ceramidase	CcLG02:15573029:15580287:1	T
	cca-miR167g	xyloglucan glycosyltransferase 4	Scaffold130637:32383:35122:1	T
169	cca-miR169i	auxin-independent growth promoter protein	CcLG01:9584030:9589068:-1	C
171	cca-miR171b	scarecrow-like protein	Scaffold000111:311133:313171:1	C
	cca-miR171k	F-box protein	Scaffold000054:76772:79124:1	C
	cca-miR171n	mitochrondrial voltage-dependent anion-selective channel	Scaffold000205:513784:517555:1	C
172	cca-miR172b	MYB transcription factor	CcLG02:33461696:33462918:-1	C
		callose synthase catalytic subunit	CcLG07:18903779:18928314:-1	C
		glycosyl transferase	CcLG07:18903779:18928314:-1	C
		callose synthase 3	Scaffold000310:304308:326141:1	C
	cca-miR172c	ethylene-responsive transcription factor	CcLG03:18195240:18197646:1	C
		floral homeotic protein APETALA 2	Scaffold132991:185259:188160:1	C
393	cca-miR393a	proline-rich protein APG	CcLG11:2735785:2737321:1	C
		zinc finger protein	CcLG11:2735785:2737321:1	C
	cca-miR393d	transport inhibitor response 1 protein	CcLG11:14918693:14921086:1	C
394	cca-miR394b	F-box protein	CcLG01:1344304:1347323:-1	C
396	cca-miR396b	WD repeat-containing protein	CcLG02:25748089:25764830:-1	C
		cytokinin dehydrogenase	Scaffold000032:188976:191574:-1	C
		peptidyl-prolyl cis-trans isomerase G	Scaffold130593:14661:23466:1	C
	cca-miR396e	growth-regulating factor	CcLG03:20414775:20417437:1	C
397	cca-miR397a	laccase/diphenol oxidase family protein	CcLG04:3250553:3253995:1	C
403	cca-miR403a	phytochrome E	CcLG06:3951623:3957773:-1	T
414	cca-miR414a	Root primordium defective protein	CcLG01:11884617:11886176:1	C
		ATP-dependent helicase	CcLG02:11525057:11530452:-1	C
		rho GTPase-activating protein	CcLG03:28864752:28867674:1	C
		beta-D-xylosidase	CcLG06:13458102:13462914:-1	C
		cytochrome P450 71A6	CcLG08:7455540:7459552:-1	C
		NAC domain-containing protein	CcLG09:8257941:8258846:-1	C
		double-strand break repair protein Mre11	CcLG10:5253626:5262725:1	C
		silencing protein 10	Scaffold125962:147152:153651:-1	C
		phosphatase 2A regulatory A subunit	Scaffold136694:16816:25260:1	C
	cca-miR414b	cyclin-D1	CcLG01:17287066:17289970:1	C
419	cca-miR419a	D-lactate/NADH dehydrogenase	CcLG01:4371421:4377385:-1	C
477	cca-miR477c	sequence independent translocase protein	CcLG02:32810955:32813695:-1	C
	cca-miR477d	WRKY transcription factor 53	CcLG10:3007026:3008574:1	C
	cca-miR477i	ATP-dependent RNA helicase	CcLG05:1409592:1419402:1	C
		eukaryotic translation initiation factor 3	CcLG11:41653935:41657857:1	C
		Unusual Floral Organs Protein	Scaffold000118:512791:514107:-1	C
		GPI mannosyltransferase 3	Scaffold130498:15029:15510:1	C
		Xyloglucan endotransglycosylase	Scaffold132287:35064:36911:1	C
		auxin response factor 9	Scaffold137159:80177:85434:-1	C
		3-beta-hydroxysteroid-dehydrogenase/decarboxylase	Scaffold126003:17943:24621:-1	T
		indole-3-acetic acid-amido synthetase	Scaffold132135:703428:705415:-1	T
	cca-miR482b	clathrin assembly protein	Scaffold135491:186958:189115:-1	C
482	cca-miR482e	peptide/nitrate transporter	CcLG11:41612792:41615296:-1	C
	cca-miR482f	arginine-tRNA ligase	CcLG08:725442:733184:1	C
821	cca-miR821c	structural maintenance of chromosomes protein 2	CcLG04:11810131:11821324:-1	C
		E3 ubiquitin-protein ligase HOS1	CcLG06:21102603:21109427:1	C
		E3 ubiquitin-protein ligase UPL1	CcLG07:6362019:6370016:1	C
		transcriptional adapter ADA2	CcLG08:15009653:15014197:-1	C
	cca-miR821d	pentatricopeptide repeat-containing protein	Scaffold132622:400224:402881:1	C
		ATP-dependent DNA helicase Q	Scaffold132470:30658:41480:1	T
829	cca-miR829b	pentatricopeptide repeat-containing protein	Scaffold135185:54668:57142:1	C
831	cca-miR831a	aminoacyl tRNA synthase	CcLG04:752969:757106:-1	C
		trigger factor protein TIG	CcLG10:13331086:13336135:1	C
		protein YLS7	CcLG11:7212783:7216281:-1	C
		3-deoxy-manno-octulosonate cytidylyltransferase	Scaffold000327:486465:492097:-1	C
		glycosyltransferase	Scaffold132135:843262:847077:-1	C

835	cca-miR835a	trichohyalin	CcLG02:16378094:16379350:1	C
837	cca-miR837a	aldose 1-epimerase family protein	CcLG11:40584336:40590799:-1	C
	cca-miR837b	transcription factor GTE1	CcLG11:13097278:13111154:1	C
		heat shock protein binding protein	CcLG11:19896716:19902951:1	C
862	cca-miR862a	rho GTPase-activating protein	Scaffold135531:38311:51604:-1	C
900	cca-miR900a	NLP4 protein	Scaffold000231:269862:274018:-1	C
902	cca-miR902a	cellulose synthase protein	Scaffold134655:13066:19257:-1	C
		cleavage and polyadenylation specificity factor	CcLG08:5553001:5580949:-1	C
		CLIP-associated protein	CcLG03:17543571:17557611:1	C
		DNA-directed RNA polymerase	Scaffold000161:43850:44041:1	C
		flocculation protein	Scaffold000372:152417:155253:-1	C
		mediator of RNA polymerase II transcription subunit 16	CcLG08:17793318:17808057:1	C
		methyltransferase PMT2	Scaffold000347:263040:266358:1	C
		polygalacturonase/glycoside hydrolase family protein	CcLG03:26708695:26711201:-1	C
		putative retrotransposon protein	Scaffold136189:143:757:1	C
		RNA polymerase beta chain	Scaffold000330:570140:570331:1	C
		secretory protein	Scaffold132952:122973:124896:1	C
		tyrosine-phosphorylation-regulated kinase 4	Scaffold136185:6650:10901:1	C
		U-box protein	CcLG01:1560356:1564234:1	C
1023	cca-miR1023b	pre-mRNA processing protein PRP39	CcLG02:26505771:26508921:-1	T
1027	cca-miR1027a	ABC transporter G family member 18	CcLG05:355370:358456:-1	C
		potassium transporter	CcLG09:2184482:2189607:1	C
	cca-miR1027b	aldehyde dehydrogenase	Scaffold133322:331285:335090:-1	C
1030	cca-miR1030a	copper-transporting ATPase	CcLG06:22426668:22434984:1	C
	cca-miR1030b	putative clathrin assembly protein	CcLG09:9384026:9392939:1	C
1078	cca-miR1078e	mitochondrial fission protein ELM1	Scaffold128870:464100:467149:-1	C
1088	cca-miR1088a	K protein	Scaffold000124:106540:106734:-1	C
1134	cca-miR1134b	GDSL esterase/lipase	Scaffold130267:548944:554966:-1	C
1435	cca-miR1435a	NAD-dependent aldehyde dehydrogenase	CcLG10:1640002:1643094:-1	C
		pentatricopeptide repeat-containing protein	Scaffold126590:136872:138020:1	C
	cca-miR1435c	7-methylxanthosine synthase	Scaffold127472:100881:112472: 2	C
		elongation factor EF-2	Scaffold129653:21989:24842:-1	T
		kinase protein	Scaffold000362:101258:104013:1	C
		kinesin-like protein KIN12B	CcLG01:1228874:1235763:1	T
		Phospholipase D/Transphosphatidylase	CcLG02:25456464:25467614:-1	C
		protein HOTHEAD	CcLG02:30769668:30771588:1	C
		protein SCO1 homolog 2	Scaffold134281:55862:58640:-1	C
		U-box domain-containing protein	CcLG07:10490493:10493800:1	C
1446	cca-miR1446a	carbon catabolite repressor protein 4	CcLG08:2887474:2891937:1	C
1507	cca-miR1507a	disease resistance protein At3g14460-like	CcLG06:21182896:21186618:1	T
		resistance protein KR4	Scaffold134207:164276:167133:1	T
1508	cca-miR1508b	pentatricopeptide repeat-containing protein	CcLG10:15216717:15221039:1	C
1510	cca-miR1510a	endo-1,4-beta-mannanase	CcLG03:26540039:26542746:-1	C
1521	cca-miR1521a	ABC transporter B family member 9/ABCB9	Scaffold127986:4869:10459:-1	C
		peroxisomal ABC transporter	Scaffold132375:20324:35909:-1	C
	cca-miR1521c	zeaxanthin epoxidase	CcLG06:18193158:18202979:1	C
		cation-chloride cotransporter	Scaffold000142:194216:218701:1	C
1522	cca-miR1522a	aldehyde dehydrogenase	CcLG08:3018563:3022692:-1	C
	cca-miR1522e	alkaline alpha-galactosidase	CcLG03:14864196:14868716:-1	C
1533	cca-miR1533a	MORN repeat-containing protein	Scaffold132391:229162:232066:-1	C
1535	cca-miR1535a	beta-galactosidase	CcLG07:1016589:1021151:1	C
		monocopper oxidase precursor family protein	CcLG11:17130562:17134309:-1	C
2055	cca-miR2055a	phospholipid/glycerol acyltransferase family protein	Scaffold135129:256685:260515:-1	C
2079	cca-miR2079a	ankyrin repeat-containing protein	CcLG03:18998712:19002905:-1	C
		exocyst complex component EXO70A1	Scaffold132955:112233:122776:1	T
2093	cca-miR2093a	chaperone activity of bc1 complex	CcLG11:41744366:41752211:1	C
		sucrose nonfermenting 4/SNF4 protein	CcLG11:46555609:46562180:-1	C
2595	cca-miR2595b	NIPA protein	CcLG10:4039818:4044783:1	C
2628	cca-miR2628a	RNA pseudouridine synthase	CcLG02:28647860:28654118:-1	C
		WAT1-related protein	CcLG08:19239178:19241414:1	T
	cca-miR2628b	E3 ubiquitin-protein ligase	CcLG03:19387849:19400066:1	C
		abscisic acid-activated protein kinase	Scaffold132224:117996:122447:-1	C
		RF1 protein (chloroplast)	Scaffold136542:14017:19494:-1	C
2655	cca-miR2655b	BPI/LBP family protein	Scaffold130267:36097:43326:1	C
		adenosine triphosphatase B subunit	Scaffold134437:122716:128312:1	C
2665	cca-miR2665a	ABC transporter B family protein	CcLG08:496829:509251:-1	C
	cca-miR2665b	Glycosyltransferase	CcLG02:15561618:15564364:-1	C
2673	cca-miR2673a	2-phytyl-1,4-beta-naphthoquinone methyltransferase	CcLG06:10532659:10535782:-1	C
		3-deoxy-manno-octulosonate cytidylyltransferase	Scaffold000327:486465:492097:-1	C
		eukaryotic translation initiation factor 3f	CcLG01:3020629:3024260:-1	C
		LRR receptor protein kinase	Scaffold000401:221975:225217:1	C

		phytol kinase 3	Scaffold132340:294171:297137:-1	C
		protoheme IX farnesyltransferase	Scaffold000310:193874:199423:-1	C
		U-box domain-containing protein	Scaffold132083:148223:150983:1	C
		UDP-N-acetylglucosamine--dolichyl-phosphate N-acetylglucosaminephosphotransferase	CcLG02:17602308:17606524:1	C
	cca-miR2673b	Acyl-CoA-binding domain-containing protein	Scaffold126139:20750:31005:1	C
		anion transporter 3	Scaffold127360:258153:262292:1	C
		cellulose synthase	Scaffold127746:232183:236297:-1	C
		glycine-rich protein	Scaffold000015:191597:192187:1	C
		mitochondrial chaperone BCS1-B	CcLG11:40175842:40177404:1	C
		osmotic stress-activated protein kinase	Scaffold126970:174571:177238:-1	C
		serine/threonine-protein kinase ndrB	CcLG08:10528707:10540651:1	C
		SPla/RYanodine receptor (SPRY) domain protein	CcLG04:137425:147491:-1	C
		Two-component response regulator ARR2 family protein	CcLG11:44913305:44916607:-1	C
2676	cca-miR2676b	alternative NAD(P)H-ubiquinone oxidoreductase C1	Scaffold130021:31887:37581:-1	T
2873	cca-miR2873a	cation/calcium exchanger	Scaffold133616:70266:72002:1	C
2931	cca-miR2931a	cysteine--tRNA ligase	CcLG03:11605341:11609429:1	C
	cca-miR2931b	glucose-6-phosphate 1-dehydrogenase	CcLG11:2682076:2686693:1	C
		serine/threonine-protein kinase	CcLG01:5693166:5703987:1	T
		cyclin-T1-4	CcLG02:32239158:32241489:-1	T
3434	cca-miR3434a	molybdopterin biosynthesis protein	CcLG03:20271123:20280574:-1	C
		6-phosphofructokinase 3	CcLG08:801502:805732:1	C
3513	cca-miR3513d	cyclic nucleotide-gated ion channel	CcLG11:541032:546042:-1	C
3629	cca-miR3629a	clathrin heavy chain	Scaffold132340:110088:122837:1	C
3950	cca-miR3950a	leucine-rich repeat family protein	CcLG07:9349543:9351744:1	C
		protein kinase	CcLG07:9349543:9351744:1	C
		beta-xylosidase/alpha-L-arabinofuranosidase	CcLG08:19034702:19039067:1	C
3951	cca-miR3951b	clathrin interactor 1	CcLG07:18185244:18186836:-1	C
3979	cca-miR3979a	FAD-linked oxidoreductase 1	Scaffold000209:71975:73564:-1	C
		fructose-1,6-bisphosphate aldolase	CcLG03:18987503:18989889:-1	C
		phosphotransfer protein	CcLG11:47160299:47161409:-1	C
		polygalacturonase	CcLG02:23963864:23971819:-1	C
		subtilisin-like protease	CcLG06:21911440:21920194:1	C
		xyloglucan endotransglucosylase	Scaffold117297:205:1251:-1	C
4245	cca-miR4245a	golgin candidate 1	Scaffold136224:32824:39987:1	C
4413	cca-miR4413b	ATP-dependent RNA helicase	Scaffold134367:16447:26548:-1	C
4415	cca-miR4415a	ascorbate oxidase	Scaffold132188:31901:39194:1	C
5031	cca-miR5031a	phosphatase	CcLG02:8302252:8305006:-1	C
		linoleate 13S-lipoxygenase 2-1	Scaffold133415:396297:402103:1	C
5057	cca-miR5057a	TSL-kinase interacting protein	Scaffold130090:30952:38177:-1	T
	cca-miR5057b	ataxin-2 homolog	CcLG10:856232:864999:-1	C
5139	cca-miR5139a	protease Do	CcLG11:26845523:26850611:1	C
5140	cca-miR5140a	tyrosine kinase family protein	CcLG06:16311492:16322056:1	C
5185	cca-miR5185b	2-alkenal reductase (NADP(+)-dependent)	Scaffold129254:35346:38243:1	C
		golgin subfamily B member	CcLG02:3438561:3444232:1	C
		ubiquitin conjugating enzyme J2	Scaffold137793:206701:209137:1	T
	cca-miR5185c	exosome complex component RRP4	CcLG03:16273064:16274885:-1	C
5201	cca-miR5201a	alpha-amylase	Scaffold132748:156518:159521:1	T
5237	cca-miR5237a	outer membrane OMP85 family protein	CcLG10:1515557:1523519:-1	C
5240	cca-miR5240b	gibberellin 3-beta-dioxygenase	CcLG01:16162168:16163492:1	C
		kinesin-like protein KIN12B	CcLG10:13553584:13559724:-1	C
5257	cca-miR5257a	plastidic glucose transporter 2	CcLG06:19509653:19514823:-1	C
5264	cca-miR5264a	chaperonin containing t-complex protein	Scaffold130382:156006:160943:-1	C
		galactinol--sucrose galactosyltransferase	CcLG11:10790201:10794069:-1	C
		peroxidase	CcLG03:15778822:15780866:1	C
5265	cca-miR5265a	polygalacturonase	CcLG08:13914669:13916836:1	T
		somatic embryogenesis receptor kinase	Scaffold000116:238231:244745:-1	T
5369	cca-miR5369a	ATP-dependent RNA helicase	Scaffold000123:182048:186102:-1	C
		auxin influx carrier protein	CcLG11:1015047:1020697:1	C
		dehydrogenase/reductase SDR family member	CcLG03:8256591:8262745:-1	C
		double-strand break repair protein Mre11	CcLG10:5253626:5262725:1	T
		LRR receptor kinase	CcLG01:1777521:1783875:1	C
		probable anion transporter	Scaffold133847:81097:82392:1	C
		proteasome assembly chaperone	CcLG06:9580481:9583958:1	C
		sucrose-phosphate synthase family protein	CcLG02:14038336:14043740:1	C
		sugar transport protein	CcLG06:22166309:22168173:1	T
		ubiquitin carboxyl-terminal hydrolase	CcLG10:7081287:7092472:1	C
		UDP-galactose/UDP-glucose transporter	CcLG04:2654307:2659352:-1	C
5382	cca-miR5382a	ADH-like UDP-glucose dehydrogenase	Scaffold000277:115342:118954:1	C
		calmodulin-domain kinase CDPK protein	CcLG02:17066188:17069151:1	C

		casein kinase	CcLG08:12834647:12840209:-1	T
		cellulose synthase protein	CcLG10:12769748:12777580:-1	C
		coatomer delta subunit	CcLG04:3746233:3751099:1	C
		cyclic nucleotide-gated ion channel 4	CcLG10:5228541:5237548:-1	T
		E3 ubiquitin-protein ligase XBAT31	CcLG10:2079989:2082656:-1	C
		endo-1,3;1,4-beta-D-glucanase	CcLG11:47431665:47435640:-1	C
		exocyst complex component SEC15B	Scaffold127746:484514:486910:1	C
		formyltetrahydrofolate deformylase	CcLG11:16832787:16836268:1	C
		inositol pentakisphosphate 2-kinase	Scaffold136554:266300:269661:-1	C
		LEC14B homolog	CcLG02:8158076:8162678:1	C
		MATE efflux family protein	Scaffold133201:166284:171831:-1	C
		monothiol glutaredoxin-S2 protein	CcLG08:17036326:17036634:1	C
		Serine/Threonine-kinase	Scaffold135298:369518:371066:-1	C
		vacuolar protein sorting-associated protein	CcLG04:7149224:7160085:1	C
		zinc-binding alcohol dehydrogenase family protein	Scaffold000277:115342:118954:1	C
5523	cca-miR5523a	apocytochrome b6	CcLG10:14146632:14147042:1	C
5721	cca-miR5721a	glutamate dehydrogenase	CcLG11:2502294:2509795:1	C
		NADP-specific glutamate dehydrogenase	CcLG10:299207:307943:-1	C
		phospholipid-transporting ATPase	Scaffold129818:388693:399035:1	C
	cca-miR5721b	dynamin-related protein 3A	CcLG09:9091914:9101513:1	T
		transcriptional regulator ATRX	CcLG02:26474610:26501820:1	C
5770	cca-miR5770a	copper amino oxidase	CcLG11:47344011:47346871:-1	C
6034	cca-miR6034a	phosphate carrier protein	CcLG11:15770855:15773041:1	C
6169	cca-miR6169a	mannosyl-oligosaccharide 1,2-alpha-mannosidase	Scaffold000063:150956:157506:-1	T
	cca-miR6169b	transcription factor bHLH48	CcLG11:39402312:39408124:-1	C
6196	cca-miR6196a	metal transporter Nramp2	CcLG03:14090772:14093942:1	C
		pre-mRNA-splicing factor ISY1	CcLG10:14898953:14899855:-1	C
6202	cca-miR6202a	26S proteasome regulatory subunit RPN13	CcLG05:1460314:1467699:1	C
		elongation defective 1 protein/ELD1 protein	Scaffold000254:66943:73616:-1	T
6232	cca-miR6232b	TFIIH basal transcription factor complex subunit	CcLG09:4004287:4015063:1	C
6281	cca-miR6281a	lysine-tRNA ligase	Scaffold000401:243611:245030:1	C
	cca-miR6281b	15-cis-zeta-carotene isomerase	CcLG01:1295933:1299525:1	C
6288	cca-miR6288e	pre-mRNA processing protein PRP39	CcLG02:26505771:26508921:-1	C
6464	cca-miR6464b	GDSL esterase/lipase At2g04570	CcLG02:12004369:12024990:1	C
6470	cca-miR6470a	sugar transport protein 5	CcLG02:5765115:5768640:1	C
7535	cca-miR7535b	DEAD-box ATP-dependent RNA helicase 29	Scaffold128595:3356:9618:1	C
		GDSL-like lipase/acylhydrolase	CcLG11:44545369:44546781:-1	C
		MLO protein	Scaffold127160:11749:18351:1	C
		NPH3 family protein	CcLG03:22579001:22582747:-1	C
		pentatricopeptide repeat-containing protein	CcLG09:78147:81913:-1	C
		RIN4c protein	Scaffold000028:144509:145446:1	C
		sucrose synthase 3	Scaffold127746:354477:359622:1	C
		WD repeat-containing protein 36	CcLG08:374039:382811:-1	C
7540	cca-miR7540a	isoleucine--tRNA ligase	CcLG04:1667045:1682520:-1	C
7545	cca-miR7545a	BPI/LBP family protein	Scaffold130267:36097:43326:1	C
		DNA helicase INO80	CcLG02:17740204:17756476:1	T
		pentatricopeptide repeat containing protein	CcLG02:15111854:15114454:-1	C
7699	cca-miR7699b	chromatin remodeling factor	CcLG02:32342892:32352993:-1	C
7736	cca-miR7736a	polygalacturonase/glycoside hydrolase family protein	CcLG11:41942310:41946957:-1	C
7776	cca-miR7776b	non-specific phospholipase C4	CcLG03:4388024:4390367:1	C
8040	cca-miR8040a	1-aminocyclopropane-1-carboxylate oxidase	CcLG10:18885090:18887441:-1	C
8041	cca-miR8041c	cyclic nucleotide-gated channel C family protein	CcLG02:1052915:1058746:1	T
8123	cca-miR8123a	2-deoxymugineic-acid 2-dioxygenase protein	CcLG02:11197795:11200386:1	C
		40S ribosomal protein S3	CcLG02:21559501:21561204:1	C
		40S ribosomal protein S8	Scaffold000287:357780:360103:1	C
		ARM repeat protein	CcLG02:32278504:32285529:1	C
		aspartokinase-homoserine dehydrogenase	CcLG11:24215052:24225901:1	T
		auxin efflux carrier	CcLG06:4104775:4108094:-1	C
		chaperone protein ClpD	CcLG03:4123233:4130463:-1	C
		chytochrome oxidase subunit 1	Scaffold128325:14609:16192:1	C
		divalent metal transporter 1	Scaffold132135:685373:688399:-1	C
		D-lactate dehydrognease 2	CcLG06:18852818:18862993:-1	C
		DUF642 family protein	CcLG03:23267975:23269703:-1	C
		elongation factor EF-2	Scaffold126898:492690:495473:-1	C
		exocyst complex component EXO70A1	CcLG02:16898494:16912381:-1	C
		exosome complex exonuclease RRP41	Scaffold000329:81511:83769:-1	C
		glutamate decarboxylase	Scaffold128736:97823:103613:-1	C
		inositol or phosphatidylinositol kinase	CcLG06:18420408:18448990:-1	C
		linoleate 9S-lipoxygenase	CcLG02:25046692:25052592:-1	C
		LRR receptor kinase	CcLG01:1777521:1783875:1	C
		LRR ribonuclease inhibitor domain protein	CcLG06:11621481:11629072:-1	C

		MDR-like p-glycoprotein	Scaffold000388:122264:131684:-1	C
		Meiotic coiled-coil protein	Scaffold134084:5282:7548:1	C
		Na+/H+ antiporter	CcLG02:35240099:35245268:-1	C
		pectin methyltransferase QUA2	Scaffold000111:20270:27658:-1	T
		plant/F20M13-60 protein	CcLG05:2468775:2474206:1	C
		plastidal glycolate/glycerate translocator 1	CcLG11:5885953:5890268:-1	C
		polygalacturonase	CcLG11:44019815:44026363:-1	C
		potassium transporter 10	CcLG08:11736547:11749120:1	C
		protein kinase ANXUR2	CcLG03:27272540:27274891:-1	T
		pumilio homolog 5	CcLG08:12253455:12259773:-1	C
		Root Primordium Defective protein 1	Scaffold000350:95394:96590:-1	C
		serine/threonine-protein kinase	CcLG11:35397402:35399557:-1	T
		U1 small nuclear ribonucleoprotein C	CcLG03:24484247:24486312:-1	C
		ubiquitin-protein ligase	Scaffold137753:42638:58020:-1	C
		vascular associated death 1/VAD1	CcLG10:5989092:6003860:-1	C
