# Supplementary material for: Genome-wide identification of miRNAs and lncRNAs in Cajanus cajan
Source: BMC Genomics. 2017 Nov 15;18:878. doi: 10.1186/s12864-017-4232-2 (PMC5688659; doi:10.1186/s12864-017-4232-2)
Supplement: Supplementary file 3 — miRNAs targeting lncRNAs in C. cajan. (PDF 46 kb) [file 12864_2017_4232_MOESM3_ESM.pdf]

Table S3: miRNAs targeting lncRNAs in *C. cajan*.

miRNA	lncRNA	Mode of action[a]
cca-miR1027a	cca-lnc-017006	T
cca-miR1030a	cca-lnc-019164	C
cca-miR1046a	cca-lnc-005268	C
cca-miR1171a	cca-lnc-020705	C
cca-miR1171a	cca-lnc-013530	C
cca-miR1217a	cca-lnc-013938	C
cca-miR1435a	cca-lnc-012596	C
cca-miR1435b	cca-lnc-008186	C
cca-miR1516c	cca-lnc-015713	C
cca-miR1522a	cca-lnc-020347	T
cca-miR1522d	cca-lnc-019372	C
cca-miR1525b	cca-lnc-012722	T
cca-miR1527a	cca-lnc-003780	C
cca-miR1527a	cca-lnc-003780	C
cca-miR1527a	cca-lnc-012486	C
cca-miR1527a	cca-lnc-011210	C
cca-miR1527b	cca-lnc-017886	C
cca-miR1533a	cca-lnc-002755	C
cca-miR1535a	cca-lnc-020033	C
cca-miR1535a	cca-lnc-008782	C
cca-miR156e	cca-lnc-013475	C
cca-miR158b	cca-lnc-020134	C
cca-miR158b	cca-lnc-016000	T
cca-miR166k	cca-lnc-019665	T
cca-miR172b	cca-lnc-017211	C
cca-miR172b	cca-lnc-011665	C
cca-miR2086a	cca-lnc-008610	C
cca-miR2595b	cca-lnc-015903	T
cca-miR2628a	cca-lnc-017394	C
cca-miR2655b	cca-lnc-006786	C
cca-miR2673a	cca-lnc-020793	C
cca-miR2676c	cca-lnc-020471	C
cca-miR2871a	cca-lnc-012842	T
cca-miR2873a	cca-lnc-020153	C
cca-miR2928a	cca-lnc-016198	C
cca-miR3979a	cca-lnc-020255	C
cca-miR3979a	cca-lnc-017415	C
cca-miR3979a	cca-lnc-013326	C
cca-miR3979a	cca-lnc-019676	T
cca-miR400a	cca-lnc-017788	C
cca-miR403a	cca-lnc-018818	C
cca-miR403a	cca-lnc-018612	C

cca-miR403a	cca-lnc-017177	T
cca-miR408a	cca-lnc-015064	C
cca-miR408a	cca-lnc-011014	C
cca-miR414b	cca-lnc-019797	C
cca-miR419a	cca-lnc-010405	C
cca-miR4233a	cca-lnc-016911	T
cca-miR4233a	cca-lnc-017063	C
cca-miR4415a	cca-lnc-020197	C
cca-miR477i	cca-lnc-014684	C
cca-miR482a	cca-lnc-012486	C
cca-miR482f	cca-lnc-012915	C
cca-miR5031c	cca-lnc-017254	C
cca-miR5054b	cca-lnc-013756	C
cca-miR5054b	cca-lnc-016844	C
cca-miR5057b	cca-lnc-011832	C
cca-miR5139a	cca-lnc-011400	C
cca-miR5171a	cca-lnc-013581	C
cca-miR5201a	cca-lnc-017751	C
cca-miR5369a	cca-lnc-013037	T
cca-miR5369a	cca-lnc-008186	T
cca-miR5565a	cca-lnc-020693	C
cca-miR6034a	cca-lnc-016088	C
cca-miR6135a	cca-lnc-017621	C
cca-miR6218b	cca-lnc-015007	T
cca-miR6281a	cca-lnc-012181	C
cca-miR6281a	cca-lnc-019771	C
cca-miR6288b	cca-lnc-017689	C
cca-miR7508a	cca-lnc-019354	C
cca-miR7535a	cca-lnc-015025	C
cca-miR7535b	cca-lnc-011922	C
cca-miR8005b	cca-lnc-020078	T
cca-miR8005b	cca-lnc-012903	T
cca-miR8041c	cca-lnc-010853	T
cca-miR8123a	cca-lnc-016125	C
cca-miR8123a	cca-lnc-013395	C
cca-miR8123a	cca-lnc-015310	C
cca-miR821c	cca-lnc-018892	C
cca-miR829c	cca-lnc-007774	T
cca-miR845a	cca-lnc-016517	C
cca-miR845f	cca-lnc-020453	C
cca-miR845g	cca-lnc-012311	C
cca-miR854a	cca-lnc-017321	T
cca-miR854a	cca-lnc-011677	T
cca-miR859b	cca-lnc-008709	C

cca-miR902a	cca-lnc-015923	C
cca-miR902a	cca-lnc-020282	C
cca-miR902a	cca-lnc-018680	T
cca-miR902a	cca-lnc-016568	T

[a]The mechanism of target inactivation: Cleavage(C) and Translational (T).
